# A mosaic influenza virus-like particles vaccine provides broad humoral and cellular immune responses against influenza A viruses

**DOI:** 10.1038/s41541-023-00728-5

**Published:** 2023-09-07

**Authors:** Xuejie Liu, Tianyi Zhao, Liangliang Wang, Zhuolin Yang, Chuming Luo, Minchao Li, Huanle Luo, Caijun Sun, Huacheng Yan, Yuelong Shu

**Affiliations:** 1 School of Public Health (Shenzhen), Sun Yat-sen University, 518107 Shenzhen, China; 2 Center for Disease Control and Prevention of Southern Military Theatre, 510610 Guangzhou, China; 3 Institute of Pathogen Biology, Chinese Academy of Medical Sciences & Peking Union Medical College, 100730 Beijing, China

**Keywords:** Vaccines, Influenza virus

## Abstract

The development of a universal influenza vaccine to elicit broad immune responses is essential in reducing disease burden and pandemic impact. In this study, the mosaic vaccine design strategy and genetic algorithms were utilized to optimize the seasonal influenza A virus (H1N1, H3N2) hemagglutinin (HA) and neuraminidase (NA) antigens, which also contain most potential T-cell epitopes. These mosaic immunogens were then expressed as virus-like particles (VLPs) using the baculovirus expression system. The immunogenicity and protection effectiveness of the mosaic VLPs were compared to the commercial quadrivalent inactivated influenza vaccine (QIV) in the mice model. Strong cross-reactive antibody responses were observed in mice following two doses of vaccination with the mosaic VLPs, with HI titers higher than 40 in 15 of 16 tested strains as opposed to limited cross HI antibody levels with QIV vaccination. After a single vaccination, mice also show a stronger level of cross-reactive antibody responses than the QIV. The QIV vaccinations only elicited NI antibodies to a small number of vaccine strains, and not even strong NI antibodies to its corresponding vaccine components. In contrast, the mosaic VLPs caused robust NI antibodies to all tested seasonal influenza virus vaccine strains. Here, we demonstrated the mosaic vaccines induces stronger cross-reactive antibodies and robust more T-cell responses compared to the QIV. The mosaic VLPs also provided protection against challenges with ancestral influenza A viruses of both H1 and H3 subtypes. These findings indicated that the mosaic VLPs were a promising strategy for developing a broad influenza vaccine in future.

## Introduction

Influenza (flu) is a contagious respiratory disease caused by influenza virus infection. Both seasonal influenza and influenza pandemics can cause serious diseases and even death, resulting in a severe economic and disease burden^1^. The most disastrous influenza pandemic in history occurred in 1918, the so-called Spanish pandemic, which has caused ~50 million deaths worldwide followed by continuous seasonal influenza epidemics and periodic pandemic threats in the past several decades^2^. Vaccination is the best intervention to prevent influenza by decreasing the infection rate and disease severity. Current seasonal vaccines are mainly classified as inactivated influenza vaccine (IIV), live attenuated influenza vaccine (LAIV), and recombinant influenza vaccine (RIV), all of which include three or four specific vaccine strains. The US Centers for Disease Control and Prevention (CDC) reported that the overall effectiveness of the influenza vaccine reached 40% in 2018–2019, which prevented approximately several million cases of illness even though there was an antigenic drift of the H3N2 strain during the season^3^. Notably, influenza vaccination provides better protection for the elderly and those with underlying medication conditions, significantly reducing the all-cause mortality rate^4^. However, influenza virus genes are composed of segmented negative-stranded RNA and are highly susceptible to antigenic drift and antigenic shift, causing unstable protection of the vaccination^5^. Vaccines are prepared each year in countries around the world using WHO-predicted pandemic strains, and influenza vaccines are highly effective when the strains match the pandemic strains, while the protection rate of influenza vaccines decreases to varying degrees when the strains do not match. Vaccination is the most effective way to prevent influenza, but current vaccination strategies have limitations. Seasonal vaccines, especially IIVs rely on neutralizing anti-HA antibodies, which only protect against similar strains and need updating annually due to antigenic variation. Production cycles are long and complicated with limited capacity, making them inadequate for pandemics. To address the limitations of current vaccine strategies and platforms, we would like to point out that there is an urgent need to optimize them and develop universal influenza vaccines.

The HA and NA viral glycoproteins are the primary targets of influenza vaccines and have garnered much interest as potential universal vaccine candidates. Some of these candidates have entered the clinical trials^6–9^.

The haemagglutinin HA is the most important antigen in influenza vaccines, triggering an immune response and producing antibodies to neutralize the virus. The induction of a broad range of HI and neutralizing antibodies against the structural domains of the HA head and stem is an attractive target for the development of universal influenza vaccines. Vaccines that induce a broad range of antibodies are being developed as new universal influenza vaccines in various countries^10–13^. In addition, NA exhibits a slower rate of antigenic drift, which makes its antigenic sites more conservative, and antibodies to NA often show a broader cross-talk^14^, so optimizing influenza vaccines to improve the targeting of NA, and thus to protect different influenza strains consistently and broadly, is becoming a new direction in the development of universal influenza vaccines.

The Mosaic Vaccine Designer will generate candidate vaccine protein cocktails that optimize the coverage, by a small set of mosaic proteins that could be included in a vaccine cocktail, of potential T-cell epitopes in a large diverse set of proteins. The mosaic algorithm has been used to predict therapeutic HIV vaccine candidates^15^ and has shown promising potential in vivo as a Pan-Filovirus vaccine. In 2014, Kamlangdee et al.^16^ constructed the first recombinant Ankara poxvirus vaccine expressing H5N1 Mosaic HA using the Mosaic approach. The results showed that the vaccine produced 100% protection against H5 AIV, while eliciting a cross-immune response to H1N1 virus in rhesus monkeys and contributing to the early in vivo clearance of the heterotypic H1N1 virus. In 2021, Bullard’s team^17^ used the Mosaic approach to design a universal swine H3 influenza vaccine. In mice, H3M vaccination resulted in significant cross-reactivity to various antibodies and T-cell responses, with higher levels of cross-reactive antibodies produced in pigs. In this study, we developed a mosaic vaccine design strategy and genetic algorithms to optimize the HA and NA sequences of human seasonal influenza viruses (H1N1 and H3N2), and we created a HA and NA mosaic cocktail containing the majority of potential T-cell epitopes of seasonal influenza viruses in order to develop a “universal” influenza vaccine. This mosaic VLPs elicits a cross-reactive humoral and cell-mediated immune response in mice models.

## Results

### Design and characterization of mosaic VLPs

**Fig. 1:**
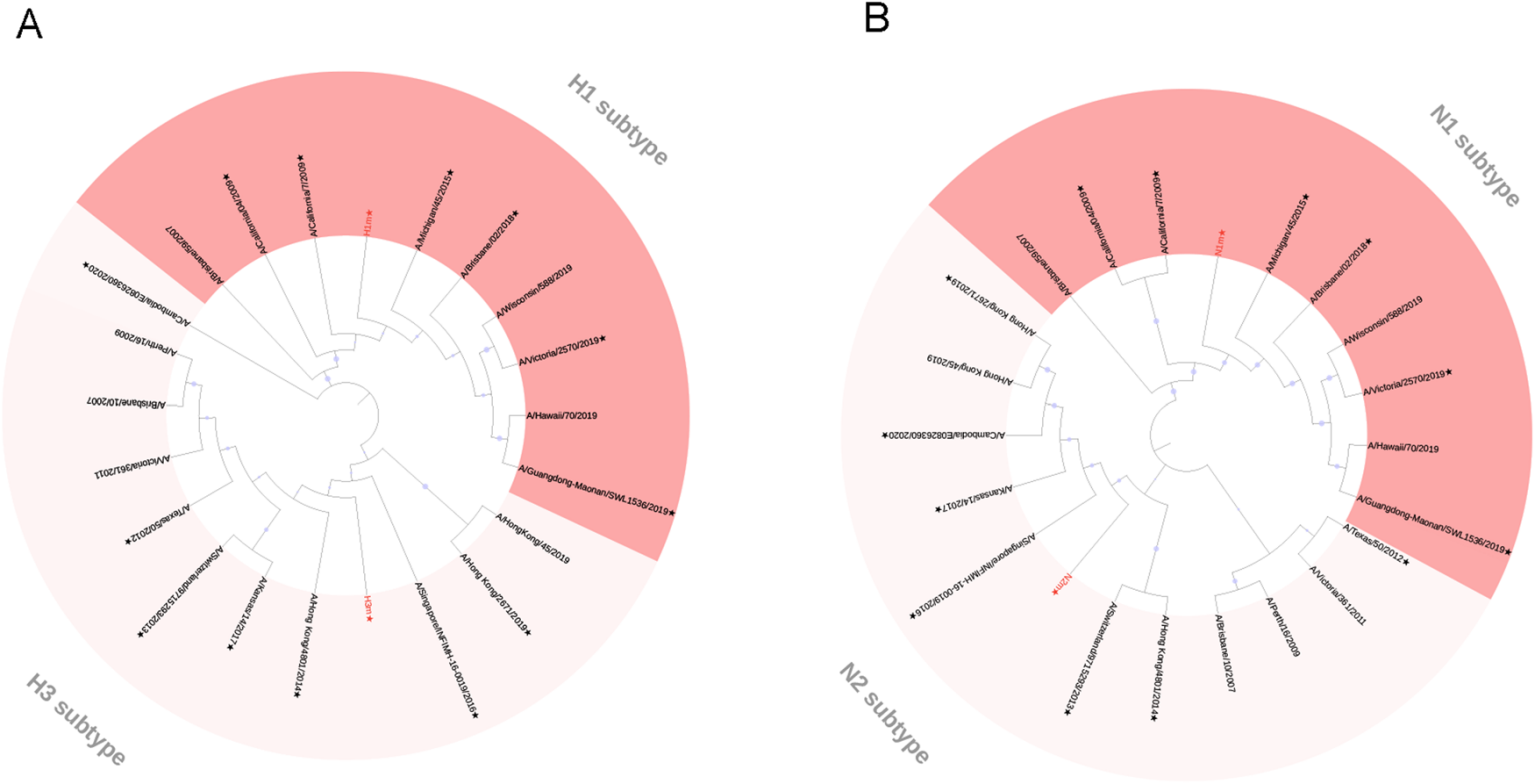
Phylogenetic relationship of the last decade of seasonal influenza virus vaccine strains hemagglutinin (HA), neuraminidase (NA) proteins, and mosaic recombinant antigens. Four mosaic recombinant antigens (H1m, H3m, N1m, and N2m) are marked by red font. H1 (red), H3(pink) virus (**A**) and N1 (red), N2 (pink) virus (**B**) phylogenic groups are indicated. Representative strains for each HA and NA subtype were aligned using ClustalW alignment and a maximum-likelihood phylogenetic tree was constructed using iTOL. An asterisk indicates inclusion in this study.

The basic principle of the mosaic antigen design strategy is to maximize coverage of potential T-cell epitopes found in circulating strains^15,18^. We have obtained four different Mosaic HA and NA amino acid sequences of H1N1 and H3N2 as described previously via the genetic algorithm of the Mosaic vaccine design strategy^13^, named H1m, H3m, N1m, and N2m (Supplementary Data), respectively. A maximum-likelihood tree was constructed to evaluate the relationship between these four mosaic immunogens and seasonal influenza virus vaccine strains from 2009 to 2022. The resulting full-length human mosaic HA and NA was analyzed based on their phylogeny and structure (Fig. 1). The identity and similarity of HA and NA amino acid sequences of strains used in this study with the mosaic HA and NA were analyzed (Supplementary Table 1).

**Fig. 2:**
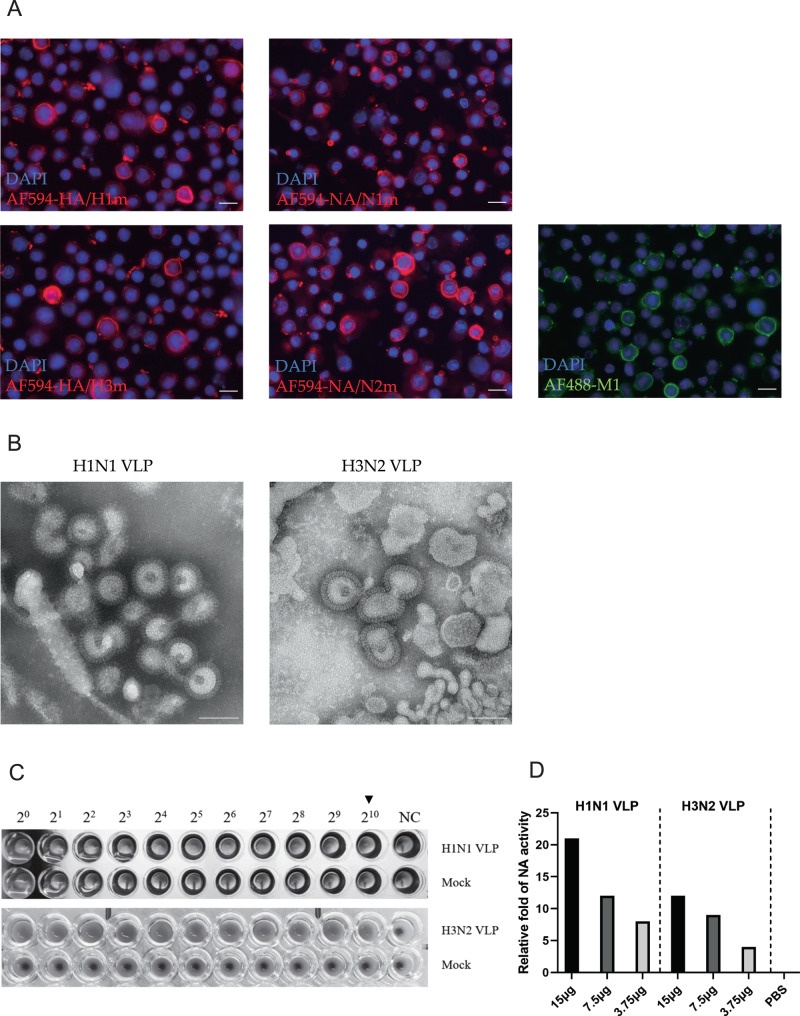
Generation and characterization of mosaic VLPs. **A** Recombinant baculovirus (rBVs) rBac-H1m, rBac-N1m, rBac-H3m, rBac-N2m, and rBac-M1 were successfully identified using immunofluorescence assay (IFA) by HA, NA, and M1-specific antibody in Sf9 cells (×200). The scale bar indicates 20 μm. **B** The morphology of VLPs similar to natural influenza virions were successfully observed by transmission electron microscopy (TEM). The scale bar indicates 100 nm. **C** The VLPs could produce 2^10 HA titers, while no HA titer was observed in Mock control group. **D** Different concentrations of VLPs had relatively high neuraminidase activity, and the neuraminidase activity increased with the increase of VLPs concentration.

To produce the mosaic VLPs, the mosaic HA and NA genes, together with the M1 gene from A/Puerto Rico/8/34 (PR8) strain were cloned into pFastBac vectors and transfected to Sf9 cells to generate recombinant baculoviruses (rBVs). The expression of the HAm, NAm, and M1 proteins was confirmed by Immunofluorescence assay. The red Immunofluorescence indicates successful protein expression of HA or NA and the green Immunofluorescence indicates successful protein expression of M1 by the respective recombinant baculovirus (Fig. 2A). Transmission electron microscopy (TEM) showed that the VLPs had irregular spikes on their surfaces, which is characteristic of influenza virus HA and NA proteins on virus particles (Fig. 2B). As HA and NA are primary protein that determines the antigenic signature and virulence of influenza viruses, we applied an HA assay to determine HA titer and found the concentrated VLPs had a titer of 2^10 (Fig. 2C). Further using standard fluorescence-based influenza neuraminidase assays, the functional NA enzyme activity of VLPs was confirmed (Fig. 2D). Quantification demonstrated the near absence of residual baculovirus DNA (≤0.65 ng/mL) in the purified mosaic VLPs (Supplementary Fig. 1). In conclusion, we successfully obtained mosaic VLPs of H1N1 and H3N2 influenza viruses. The obtained mosaic VLPs included viral HA, NA, and M1 proteins with morphological characteristics similar to those of natural viral particles.

### Mosaic VLPs elicit robust, broad-spectrum humoral antibody responses

**Fig. 3:**
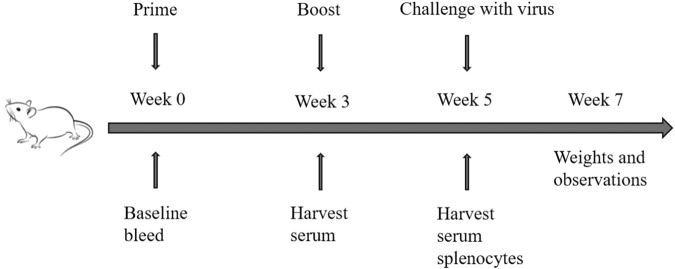
Experimental plan: vaccines, immunizations and challenge studies. Mice received a boost immunization (I.M.) with 1.5 μg of HA content of monovalent mosaic VLP vaccine or 1.5 μg of HA contents of quadrivalent inactivated influenza vaccine (QIV). Animals in the negative control group received PBS. Five weeks after vaccination, mice were intranasally (I.N.) infected with influenza virus containing 10MLD50.

**Fig. 4:**
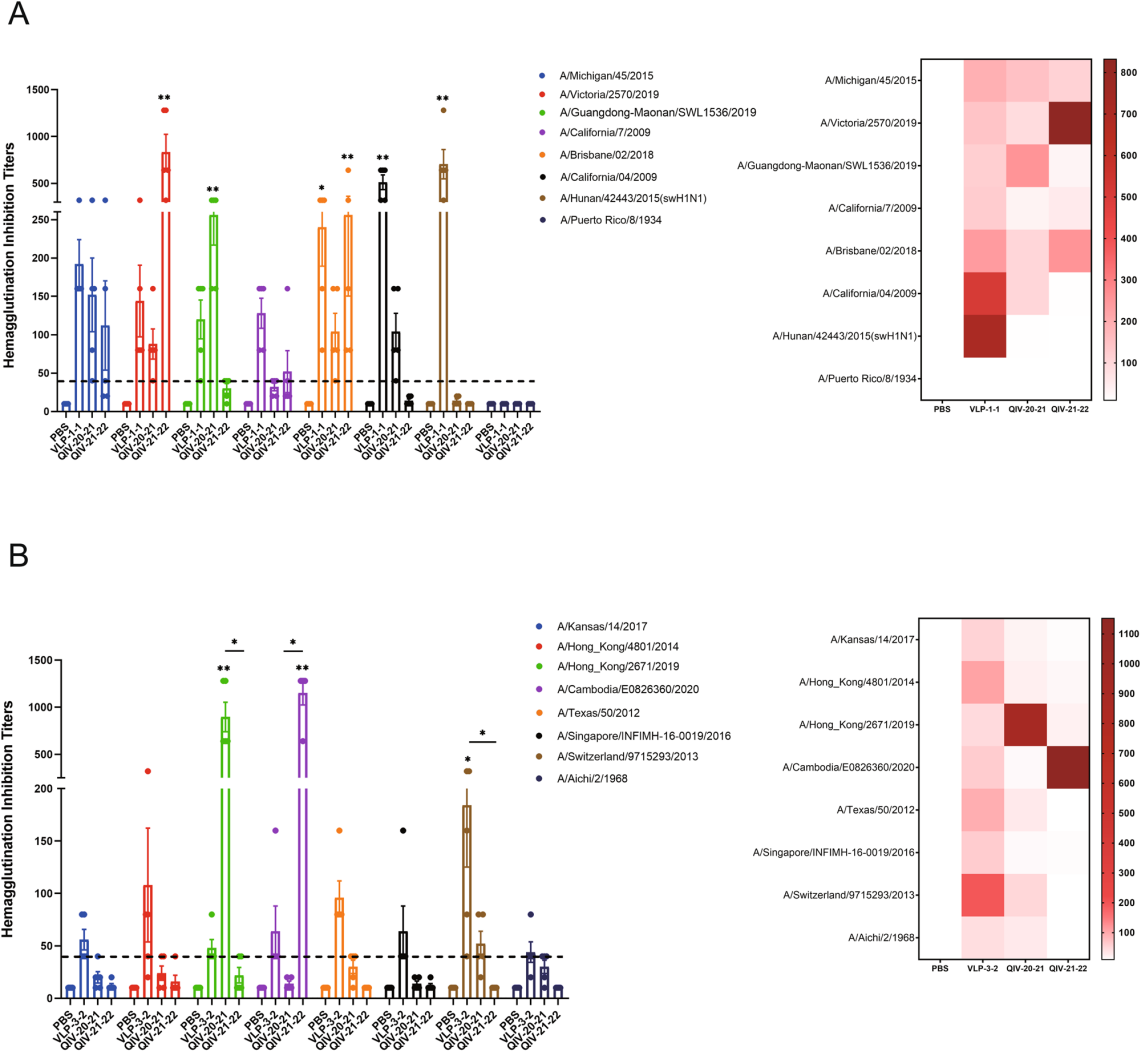
Mosaic VLPs immunization elicits robust cross-reactive antibody responses in mice against H1- and H3-representative viruses. BALB/c mice were vaccinated with VLPs and compared to QIV-vaccinated mice. The HI antibody response to H1 (**A**) or H3 (**B**) representative strains was determined by HI assay in mice immunized with boost immunization. A heatmap of HI titers was further constructed to better visualize cross-reactive antibody response for each vaccine. Data are presented as the mean with standard error (SEM) (*n* = 5; two-way ANOVA with Tukey multiple comparison), *P < 0.05, **P < 0.01.

We next evaluated the immune response stimulated by the mosaic VLPs in mice. We first investigated the antibody response within 35 days after the prime-boost vaccination regimen (Fig. 3). In humans, an HI titer of at least 40 (antibody levels protecting from infection in approximately 50%, is often used as a threshold protective titer) is often used as the protection antibody titer^19^. Mice with the mosaic VLPs immunogens vaccination resulted in a robust cross-reactive antibody response, with HI titers ≥40 to 7 of the 8 (87.5%) tested H1 strains (Fig. 4A) and 8 of the 8 (100%) H3 tested strains (Fig. 4B), especially A/Hunan/42443/2015, which is a swine influenza virus, with HI titer ≥640. In contrast, the QIV provided limited cross-reactivity with mismatched strains, as shown in the heatmap (Fig. 4). This indicated that the mosaic VLPs vaccine could induce potent cross-reactive immunity against some mismatched strains. Notably, mosaic antigen showed high cross-immunity to all vaccine strains during the last decade, significantly higher than the QIV group. These promising results supported that the mosaic VLPs vaccine could protect against different seasonal influenza viruses circulating in humans over the past decade.

**Fig. 5:**
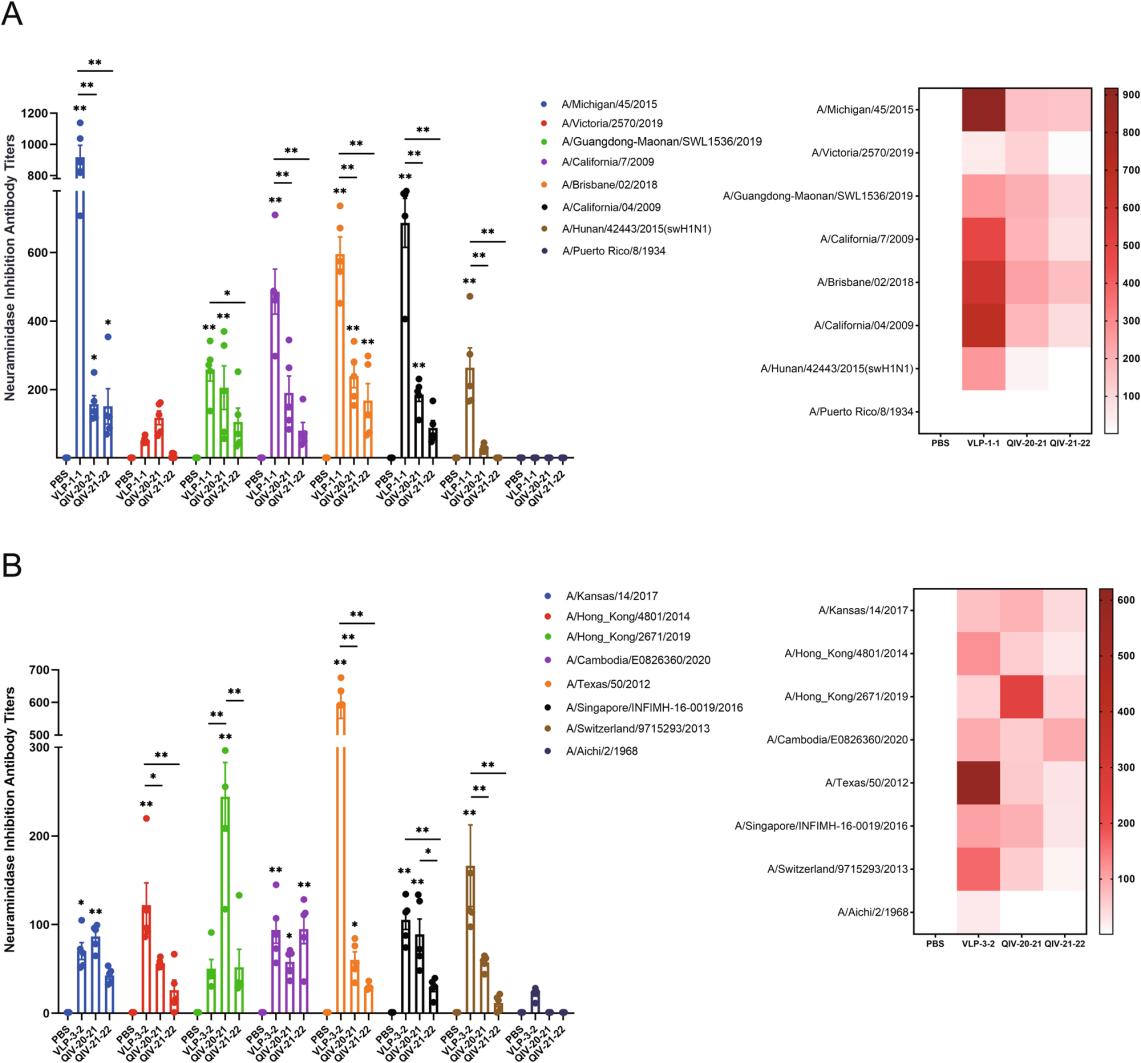
Mosaic VLPs immunization elicits robust cross-reactive antibody responses in mice against N1- and N2-representative viruses. BALB/c mice were vaccinated with VLPs and compared to QIV-vaccinated mice. The NI antibody response to N1 (**A**) or N2 (**B**) representative strains was determined by ELLA assay in mice immunized with boost immunization. A heatmap of NI titers was further constructed to better visualize cross-reactive antibody response for each vaccine. Data are presented as the mean with standard error (SEM) (*n* = 5; two-way ANOVA with Tukey multiple comparison), *P < 0.05, **P < 0.01.

To further investigate the mosaic NA immunogen response after vaccination in mice, the NA inhibition titer was evaluated by ELLA assay. The results showed that mosaic VLP-vaccinated mice produced higher titers of NI antibodies against all N1 (Fig. 5A) and N2 (Fig. 5B) subtypes of seasonal influenza virus vaccine strains involved in the study over the past decade, indicating that mosaic VLPs induced strong cross-reactive immunity. Compared with the mice immunized with the QIV, it only induced NI antibodies against a few vaccine strains and did not induce potent NI antibodies against their components. In particular, the 2021–2022 QIV (QIV21-22) has no NI antibody titer against A/Victoria/2570/2019 strains, which was also related to the fact that the main component of the influenza vaccine immunogen is HA protein rather than NA protein. We quantified NA content in both QIV and mosaic VLPs to investigate whether the observed difference was attributed to different NA content in the vaccine (Supplementary Fig. 2). Our analysis revealed that N2 of 2020–2021 QIV (QIV20-21) had slightly higher NA content than VLPs, while other NA content of QIV were lower than mosaic VLPs. The heatmap demonstrated a substantial difference in NI titers between Mosaic VLPs and QIV (Fig. 5).

**Fig. 6:**
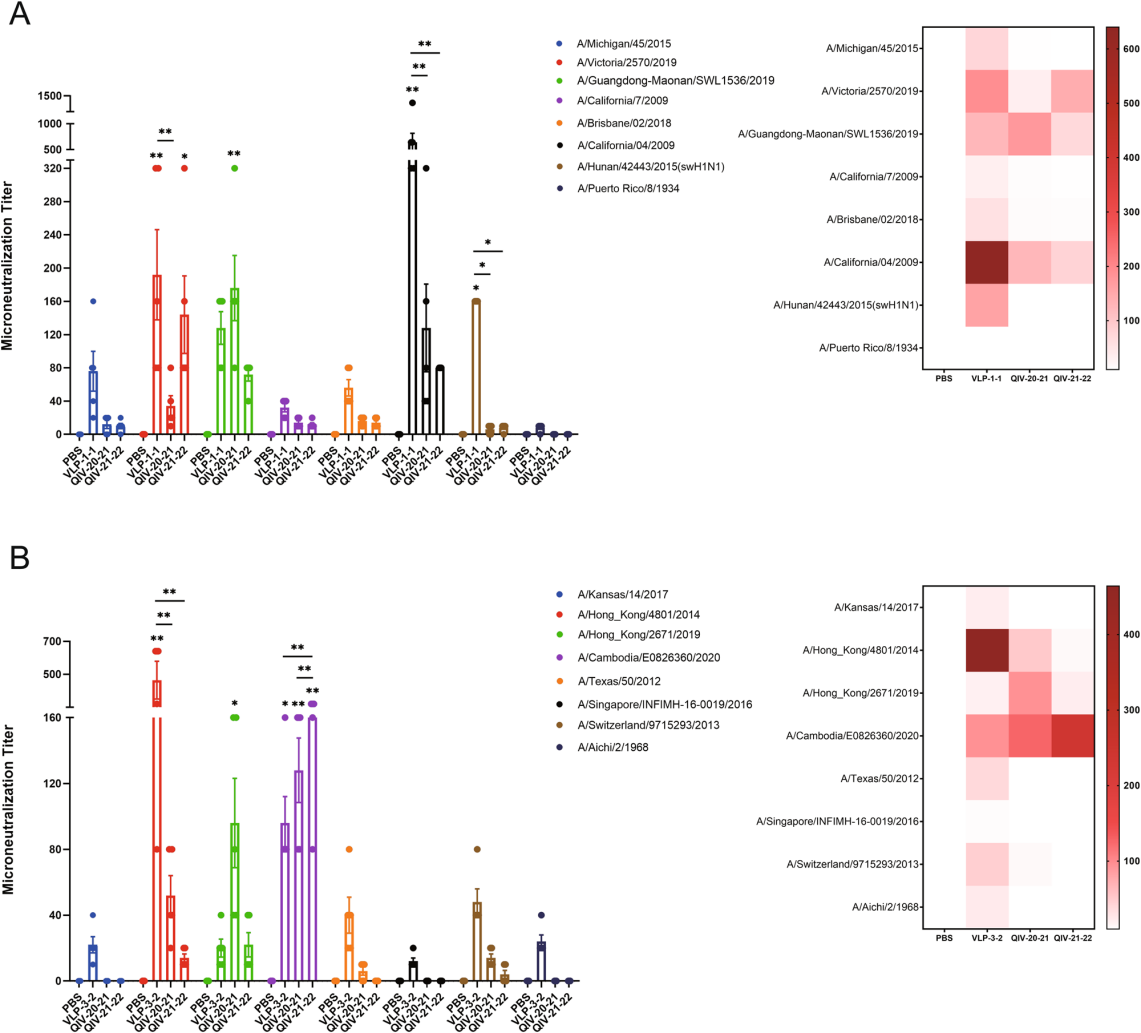
Mosaic VLPs immunization elicits cross-reactive neutralizing antibody responses in mice against H1N1- and H3N2-representative viruses. BALB/c mice were vaccinated with VLPs and compared to QIV-vaccinated mice. The neutralizing antibody response to H1N1 (**A**) or H3N2 (**B**) representative strains was determined by Microneutralization assay in mice immunized with boost immunization. A heatmap of Microneutralization titers was further constructed to better visualize cross-reactive antibody response for each vaccine. Data are presented as the mean with standard error (SEM) (*n* = 5; two-way ANOVA with Tukey multiple comparison), *P < 0.05, **P < 0.01.

We then measured neutralizing antibody titers using MDCK cells to assess the serum-neutralizing antibody levels of each vaccinated mouse. The vaccine-induced neutralizing antibody pattern was consistent with the results based on HI and NI assays, confirming the function of these cross-reactive antibodies (Fig. 6). Micro-neutralizing potency in sera from mice immunized with the mosaic VLPs vaccine was significantly higher than in the PBS group, with 1.3–44.0-fold increases in nAb titers (GMTs) compared to mice immunized with QIV for some strains, respectively. Overall, the Mosaic VLPs vaccine provided comprehensive broad-spectrum neutralizing coverage of most virus strains involved in the experiments.

The functional binding of vaccine-induced HA or NA antibodies was evaluated by HI, NI, and MN activity using various influenza viruses (Figs. 4–6 and Supplementary Fig. 1). To further investigate the correlates of protection, antibody production in vaccinated mice was evaluated. Levels of vaccine-specific IgG, IgG1, or IgG2a antibodies were measured by ELISA in serum (Supplementary Figs. 3 and 4). After the second vaccination, the mosaic VLP vaccines produced high levels of IgG antibodies against all four vaccine strains. Moreover, a strong antibody response against the historical influenza strains (PR8 and X-31) was detectable only in the group that received VLPs, with weaker responses to QIV. To assess the antibody response induced by the mosaic H1 and H3 antigens, serum obtained after vaccination was tested for their capacity to bind to the respective VLP proteins. As shown in Supplementary Fig. 4, the mosaic H1N1 VLP elicited higher antibody titers than the mosaic H3N2 VLP, indicating that the mosaic H1 antigen elicited a better antibody response than the mosaic H3 antigen. Interestingly, the mosaic VLPs induced higher IgG2a antibodies than IgG1 antibodies, while QIV induced more IgG1 antibodies. This suggested that mosaic VLPs elicit a predominantly Th1 cell-dominated immune response in vivo, whereas QIV elicits a Th2 cell-dominated immune response.

### Mosaic VLPs elicit antigen-specific cellular immune responses

To evaluate the antigen-specific T-cell responses boosted by VLPs, the splenocytes from mice post 35 days of VLPs, QIV or PBS inoculation were isolated and stimulated with inactivated H1N1 and H3N2 influenza viruses. T-cell-produced IFN-γ (T-helper 1) and IL-4 (T-helper 2) were measured by ELISpot assay. The expression of cytokines for T cells was quantified by flow cytometry.

**Fig. 7:**
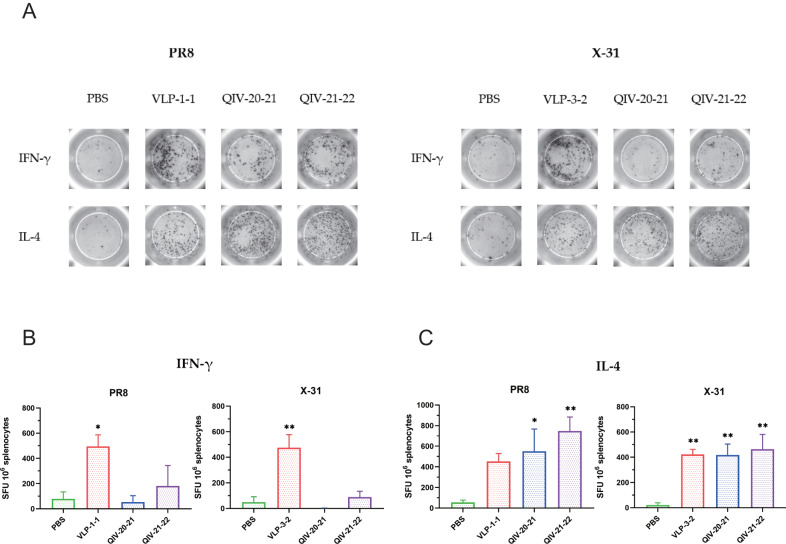
Mosaic VLPs immunization elicits T-cell responses in mice against H1N1- and H3N2-representative viruses. **A** Splenocytes isolated from immunized BALB/c mice in each group on days 35 were plated in ELISpot wells to detect IFN-γ and IL-4 cytokine-producing cells. **B**, **C** Splenocytes restimulated with inactive A/Puerto Rico/8/34 (PR8) and A/Aichi/2/1968 (X-31) virus, analyzed for cellular immunity using an IFN-γ and IL-4 ELISpot assays. Data are presented as the mean with standard error (SEM) (*n* = 5; one-way ANOVA with Tukey multiple comparison), *P < 0.05, **P < 0.01.

As shown in Fig. 7, the IFN-γ-secreting cells in the mosaic VLPs vaccine group were significantly higher than those in the PBS group, while IFN-γ secretion was reduced in the QIV group, especially in mice immunized with the QIV20-21 vaccine. The mosaic VLPs vaccine induced a strong T-helper 1-associated T-cell immune response, while the QIV did not elicit a robust antigen-specific T-cell response after vaccination as compared to the PBS (Fig. 7A). At the same time, we observed higher levels of IL-4 expression in all mosaic VLPs groups (Fig. 7B).

**Fig. 8:**
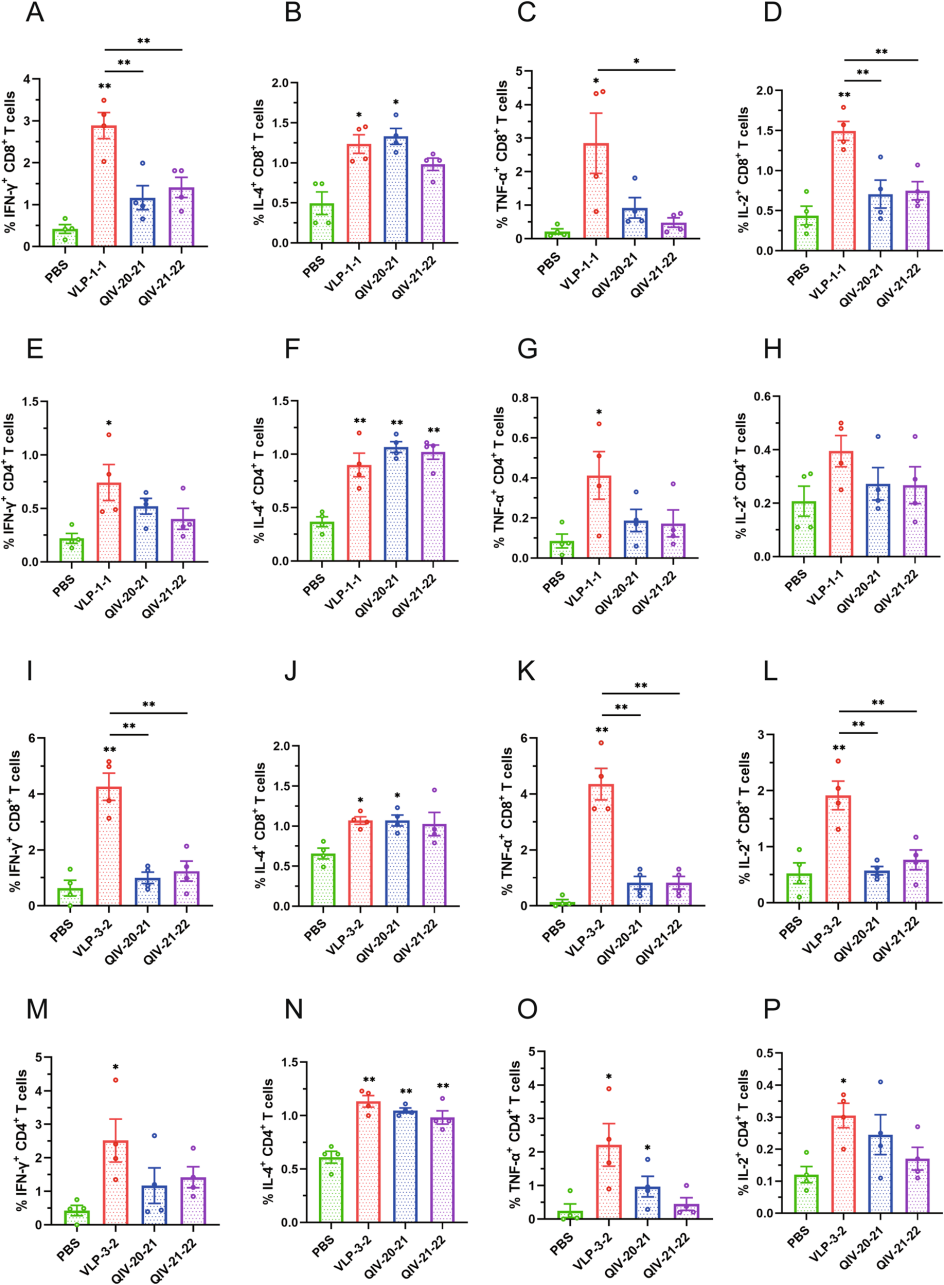
Splenocytes isolated from immunized BALB/c mice in each group on day 35, and T-cell responses were detected by flow cytometry after stimulation of the spleen with virus A/PR/8/1934 (PR8) or A/Aichi/2/1968 (X-31). Shown are detailed results of CD8^+^ IFN-γ^+^ T cells (**A**), CD8^+^ IL-4^+^ T cells (**B**), CD8^+^ TNF-α^+^ T cells (**C**), CD8^+^ IL-2^+^ T cells (**D**), CD4^+^ IFN-γ^+^ T cells (**E**), CD4^+^ IL-4^+^ T cells (**F**), CD4^+^ TNF-α^+^ T cells (**G**), and CD4^+^ IL-2^+^ T cells (**H**) induced by virus PR8 and detailed results of CD8^+^ IFN-γ^+^ T cells (**I**), CD8^+^ IL-4^+^ T cells (**J**), CD8^+^ TNF-α^+^ T cells (**K**), CD8^+^ IL-2^+^ T cells (**L**), CD4^+^ IFN-γ^+^ T cells (**M**), CD4^+^ IL-4^+^ T cells (**N**), CD4^+^ TNF-α^+^ T cells (**O**), and CD4^+^ IL-2^+^ T cells (**P**) induced by virus X-31. Data are presented as the mean with standard error (SEM) (n = 4; one-way ANOVA with Tukey multiple comparison), *P < 0.05, **P < 0.01.

We further explored the CD8^+^ and CD4^+^ T-cell response induced by the VLPs vaccine using flow cytometry. The representative flow images of CD8^+^ IFN-γ^+^, CD8^+^ IL-4^+^, CD8^+^ TNF-α^+^, CD8^+^ IL-2^+^, and CD4^+^ IFN-γ^+^, CD4^+^ IL-4^+^, CD4^+^ TNF-α^+^, CD4^+^ IL-2^+^ T cells under virus A/PR/8/1934 stimulation are shown in Supplementary Fig. 5. The gating strategies are shown in Supplementary Fig. 6. The frequencies of the antigen-specific splenocytes from the dually immunized mice were capable of releasing the cytokines IFN-γ, TNF-α and IL-2 were higher than those from QIV or PBS immunized mice, as determined by intracellular cytokine staining (Fig. 8A–H). The same results described above were observed under A/Aichi/2/1968 stimulation (Fig. 8I–P). These results showed that mosaic VLPs elicited substantial cellular immune responses after immunization. Interestingly, the mosaic VLPs vaccine induced a significant T-cell response to the PR8 virus, although it did not elicit a detectable antibody response against this virus (measured by HI and NI titers). Therefore, it is very feasible to select this strain to detect the potential of vaccine-induced T-cell response in the absence of detectable cross-reactive antibodies.

### Mosaic VLP-vaccinated mice were protected from lethal challenges with H1N1 and H3N2

To examine the protective effect of vaccination, all mice were intranasally challenged with 10 mouse-lethal doses of 50% (MLD50) of A/Puerto Rico/8/34(PR8) and A/Aichi/2/1968 (X-31) 35 days after vaccination. Following the challenge, mice were monitored for 14 consecutive days for weight loss and were humanely sacrificed if a 20% weight loss was achieved. Mice vaccinated with the placebo vaccine (PBS) lost more weight than any other group, a peak weight loss of 10% was reached at 3 days post infection (dpi).

**Fig. 9:**
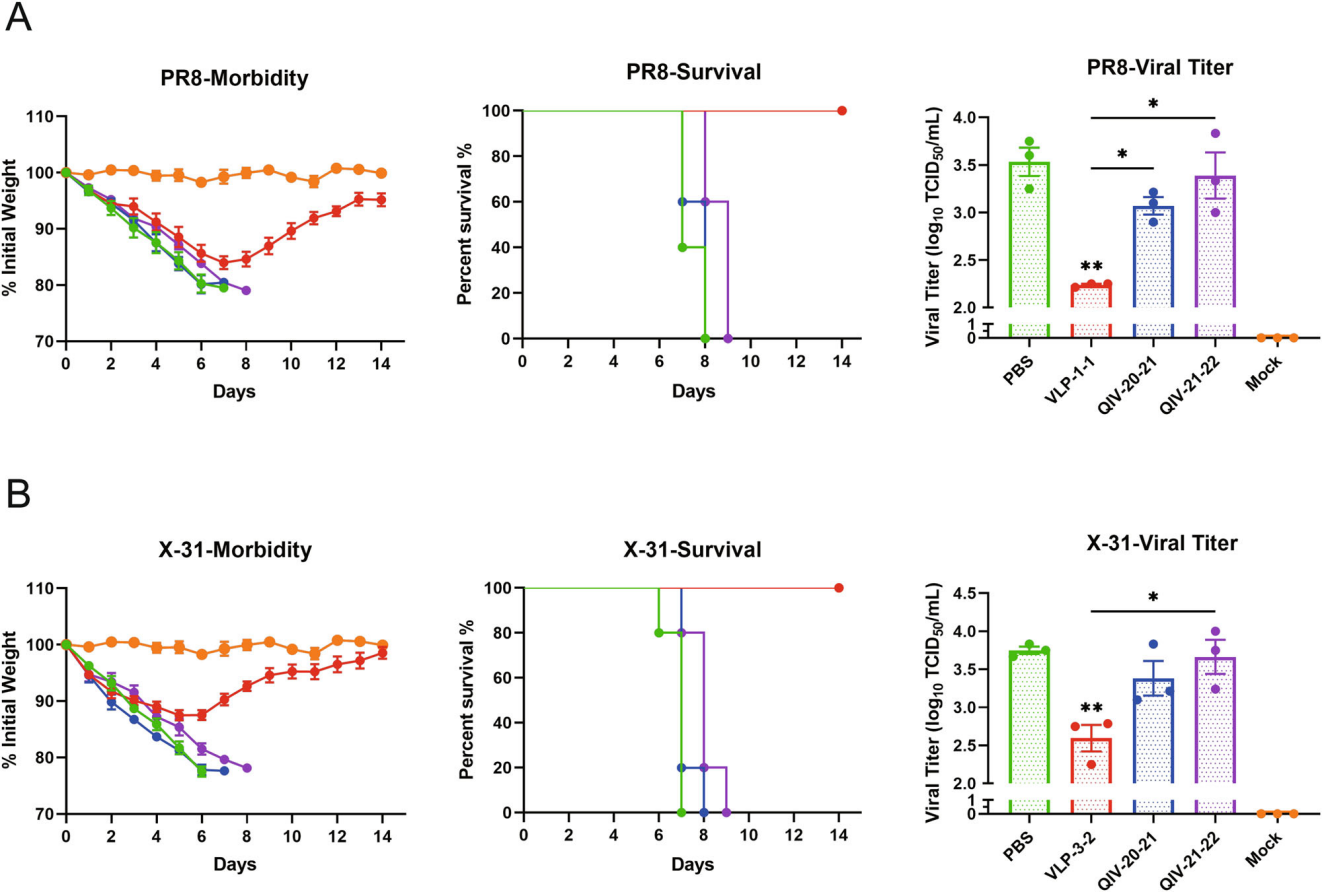
The prophylactic activity of mosaic VLPs vaccine in an influenza A virus-challenge model. At 2 weeks post boost immunization, the vaccinated mice were challenged by A/Puerto Rico/8/34 (**A**) and A/Aichi/2/1968 (**B**) viruses. Body weight loss and survival rates of mice were recorded every day after the virus challenge for 14 days (*n* = 5). Mice were sacrificed on day 4 post infection to determine lung viral titer by TCID50 assays. (*n* = 3, one-way ANOVA with Tukey multiple comparison), *P < 0.05, **P < 0.01.

This trend was also observed in all other mosaic VLPs vaccine groups, with weight loss peaking at 2 dpi but then rising steadily until 7 dpi, after which weight gain stabilized. When challenged with PR8 or X-31, mosaic VLP-vaccinated mice exhibited 100% survival and experienced only moderate weight loss before recovery (PR8 was 16% (Fig. 9A), X-31 was 12.5% (Fig. 9B), all mice receiving the QIV and PBS vaccines died after the challenge.

To evaluate the protective effects of mosaic VLPs, the viral loads in the lungs were analyzed at 4 dpi (Fig. 9). The results showed that the mosaic VLPs vaccine groups experienced significantly reduced in viral shedding by historical strains belonging to the H1 and H3 strains. The level of viral shedding in these groups at 4 dpi was significantly lower than in the PBS group (*P* < 0.001). In contrast, the QIV-vaccinated mice had similar levels of viral shedding in the lungs to those in the PBS group, reaching 10^3.07–3.66 TCID50/mL. Taken together, these data demonstrate that mosaic VLPs vaccine can offer significant protection against the lethal influenza virus challenge.

### Mosaic VLPs vaccination reduced the inflammatory response in mice lung histopathology

To further characterize the protection observed in the VLPs or QIV-vaccinated groups, we next examined hematoxylin and eosin (H&E)-stained lung sections harvested at 4 dpi (Supplementary Fig. 7). First, we observed the clinical manifestations of the mice after the challenge. As expected, control mice immunized with the PBS exhibited typical clinical signs such as shaggy hair, body tremors, depression, and loss of appetite after the challenge. Moreover, mice vaccinated with QIV also showed similar clinical signs, with less severe clinical manifestations compared to controls. In contrast, mice immunized with VLPs vaccines did not show significant clinical signs. A comparative histopathological study of the lungs from mice on day 4 after challenge with live IAV strains was performed. In PBS groups and QIV-vaccinated groups, histopathological observations revealed extensive lung tissue damage, such as alveolar wall thickening, connective tissue hyperplasia, massive alveolar detachment with hemorrhage and vascular inflammation, and massive inflammatory cell infiltration (Supplementary Fig. 7A, C, D). However, we observed reduced lung tissue damage in the lungs of mice inoculated with mosaic VLPs, which showed a lighter cellular infiltration and inflammation. In both VLPs groups, damage to the bronchiolar epithelium and signs of alveolar interstitial cell infiltration were minimal compared with PBS and QIV groups, and the presence of bronchial-associated lymphoid tissue (BALT) around the lung bronchi, a feature that contributes to lung tissue cell recovery (Supplementary Fig. 7B). Overall, histopathological evaluation of lung sections of IAV-challenged mice revealed a high degree of protection by mosaic VLPs vaccines, with evidence of slightly higher protection against pulmonary pathology in mosaic VLPs groups than in QIV groups.

## Discussion

Influenza vaccine components must be updated yearly due to the frequent antigenic drift of influenza viruses. Existing influenza vaccines have a narrow protection spectrum and short immune persistence. Therefore, there is an urgent need to improve the traditional influenza virus vaccine and develop a “universal” influenza vaccine. In this study, we evaluated mosaic vaccine design algorithms for broad cross-reactivity of H1N1 and H3N2 for HA, NA immunogen design to create a potential universal influenza A vaccine.

In this study, we demonstrate that our mosaic VLPs vaccine induced strong cross-reactivity against various IAV viruses representing the last decade of seasonal influenza viruses and some historical strains. After primary and booster immunization of mice, the mosaic VLPs vaccine induced strong HI cross-reactive antibody titers against 15 of 16 different IAV strains (Fig. 4). In contrast, the QIV showed limited cross-reactivity outside of the strains contained in each vaccine. Moreover, mosaic VLPs vaccination elicited HI antibody titers are much stronger when compared to the QIV. Although an HI titer of 40 is recognized as the threshold antibody titer that reduces the risk of influenza infection by 50%, the stronger the HI titer, the higher the level of protection^20^. In addition, higher titers are often needed to protect at-risk populations, such as pediatric or elderly populations^21,22^. Further immunization studies in mice support the excellent cross-reactive immunity induced by the mosaic VLPs vaccine. Importantly, we found that a single immunization with mosaic VLPs induces strong cross-reactive antibody titers, especially for H1N1 subtype strains, while immunity with QIV produces very limited antibodies to mismatched strains (Supplementary Fig. 8). This data shows that the mosaic VLPs can be used as a broad cross-reactive vaccine, and only one immunization may be required to induce strong immunity. Importantly, this data suggests that booster immunization is critical for generating a significant antibody response when administering the commercial vaccine QIV. At the same time, we found that the serum of mice immunized by mosaic VLPs also produced extremely high titers of HI against a swine influenza virus strain A/Hunan/42443/2015(H1N1), even far beyond other seasonal influenza vaccine strains, which suggests that the antigen epitope of our mosaic sequence covers the swine flu strain as well. It is suggested that this VLPs might provide some cross-host protection and transmission. Interestingly, we also observed the mosaic H1N1 subtype VLP vaccine induced much higher levels of cross-reactive antibodies than the mosaic H3N2 subtype VLP vaccine. On average, a twofold higher magnitude of antibody response producing with the H1 subtype than the H3 subtype, despite both antigens being injected at the same dose. This observation may indicate a degree of immune advantage of the mosaic H1 antigen over the mosaic H3 antigen. The result may be partly because the H3N2 subtype tends to change rapidly genetically and antigenically. The current H3N2 subtype has evolved to form many independent and genetically different branches, and a variety of mutant strains spread together, resulting in poorly conserved antigen epitopes and more antigen epitopes losing their function due to mutations. This is also more consistent with the conclusion of the CDC’s vaccine effectiveness observation study conducted on the general population: the vaccine has played a good role in reducing the severity of influenza A H1N1 and influenza B virus infection and outcome, but the degree of protection against Influenza A H3N2 is low^23,24^. However, the effect of heterosubtypic immunodominance among influenza virus vaccine strains has not been extensively studied, but increasing the dose of the least immunogenic HA subtype could help overcome the limitations of the immune response observed in multivalent vaccine formulations^25,26^. Therefore, in the future, if a multivalent mosaic VLPs vaccine is developed, it is also an essential task for us to determine the dose ratio of the H1 and H3 antigen mix to optimize the host immune response to both components after vaccination.

These functions of neutralization and HI antibody are significant contributors to the traditional protection mediated by seasonal influenza virus vaccines. They may also play a role in the protection provided by novel vaccination methods. We also evaluated the serum-neutralizing antibody potency in vaccinated mice accordingly. The functional nature of vaccine-induced Abs in mosaic VLPs was confirmed by the MN results, which showed an overall correlation with HI titers. Although occasional differences between HI and MN results have been reported, and there is some evidence that MN results may be a more sensitive predictor of child protection^27^, our data suggest that Abs induced after vaccination with mosaic VLPs both inhibit hemagglutination and prevent viral entry into host cells (neutralization).

Neuraminidase (NA) is the second largest surface antigen of influenza viruses, and NA immunity has been indicated as an independent correlate of protection, as demonstrated in the analysis of human samples^28^. It has been suggested as a complementary antigen to the primary immunodominant hemagglutinin (HA) antigen in influenza vaccines^29^. Compared to HA, NA is less affected by antigenic drift, induces a strong anti-neuraminidase immune response, and provides broader protection against many influenza virus strains. Many reports have shown that NA protein-induced protection is mediated by NI antibodies^14,30,31^. Therefore, one goal of developing an NA-based influenza vaccine is its ability to effectively induce NI antibodies to confer protection against influenza viruses^32^. In this study, we also demonstrated NA-based NI antibody activity and found that mosaic VLPs induced higher titers of cross-protective NA-specific antibodies in mice. The serum of mice vaccinated with mosaic VLPs can inhibit the neuraminidase activity of a variety of influenza A H1N1 and H3N2 strains (7/8 in the N1 subtype; 8/8 in the N2 subtype), while QIV were barely able to induce potent NI antibodies (Fig. 5). Importantly, mosaic VLPs induced a broader spectrum and higher titer NI antibody compared with HI antibody, which correlates with the fact that NA antigens are more conserved and less affected by antigenic drift. Previous studies have found that HA is known to be immunodominant and has a suppressive effect on inducing NA-specific immune responses when both HA and NA antigens are present in the same influenza virus particle, as in inactivated influenza vaccines^33^. Most NA inhibitory antibodies are considered to mediate non-neutralizing immunity, resulting in infectious protection but significantly reducing disease severity^34,35^. NA has also been reported to promote HA-mediated cell fusion and viral infection of target cells, indicating the role of NA in viral entry^36^. In this study, HA protein content was used as the quantitative standard for immunization dose, which made it impossible to compare the immune responses elicited by the NA antigen fraction in each vaccine because the immunized mosaic VLPs and QIV had the same HA content but different NA content. We quantified NA content in QIV and Mosaic VLPs to determine this difference. We demonstrate that mosaic VLPs can induce a broad spectrum of NI antibodies. In addition, mosaic VLPs elicited higher NI titers and broader spectrum NI antibodies, even though the N2 content in VLPs was lower than the NA content in QIV20-21, which was a very surprising finding. A shortcoming of this study is that we do not have a standardized NA for our immunogens measurement. Therefore, whether an independent NA vaccine entity is desirable would also be a direction for us to consider in future.

Since HI titers have long been the primary criterion for influenza vaccine licensing, it is unsurprising that most commercially available influenza vaccines (such as TIV or QIV) are designed to induce a robust humoral response. However, most protective responses to viral diseases arise from close cooperation between different immune system elements: innate and adaptive, humoral and cellular^37^. Although pre-formed antibodies can protect against many viral diseases, T-cell-mediated immunity is generally required to clear most viral infections and maintain long-term immunity effectively. In humans, the development of cross-reactive T cells and memory T cells is associated with lasting immunity to the influenza virus^38,39^. Moreover, a growing consensus is that T-cell-mediated immunity plays a crucial role in long-term cross-protective immunity against influenza, which is especially important in children and the elderly^40,41^. In recent years, many studies have also confirmed the critical role of cytotoxic T lymphocytes (CTL) in anti-influenza immunity and clearance of influenza viruses^38,42,43^. Both CD4^+^^44^ and CD8^+^^45^ T cells have been implicated in contributing synergistically to protection. However, with the except live attenuated vaccine (LAIV), commercial influenza vaccines usually have difficulty inducing a strong antiviral T-cell-mediated immunity^46^. The ELISpot and flow cytometry results of this study showed that mosaic VLPs could induce influenza virus antigen-specific cellular immunity in mice. Immunization of mice with mosaic VLPs vaccine induced highly cross-reactive T-cell responses to historical virulent strains. Importantly, although mosaic VLPs vaccination did not induce significant HI antibody responses to PR8 and X-31, vaccination induced high T-cell responses to both historical strains and provided complete protection after the lethal challenge. In addition, the pathological examination revealed a significant reduction in lung damage and a maximal decrease in lung virus titers, potentially leading to a decrease in viral shedding and subsequently lowering both intraspecific and interspecific transmission. Furthermore, compared to other vaccinated mice, the spleen lymphocytes of mice immunized with mosaic VLPs induce higher levels of IFN-γ, INF-α, and IL-2, all significantly higher than IL-4, highlighting the robust activation of the CTL immune pathway by our mosaic immunogen.

This phenomenon suggested that it mosaic VLPs induces strong Th1 cell-mediated immune response and inflammatory response production, while the high level of secreted IFN-γ inhibits the mediation of Th2 cell immune response. Combined with the results of the challenge protection effect experiments, it is clear that the secretion of cytokine IFN-γ, INF-α and IL-2 can protect experimental mice from a lethal dose of influenza virus. The immune response of Th2 cells can activate immune B cells, which in turn induces the body to produce a large number of antigen-specific antibodies. The antibodies neutralize pathogen molecules and thus protect the body from pathogens. The level of specific IgG2a antibodies induced by VLPs was higher than that of IgG1, as Th1 cells in mice produce IgG2a antibodies. Previous studies have found that IgG2a isotype monoclonal antibodies are more effective than IgG1 isotype antibodies at clearing virus infections^47–49^. Furthermore, the involvement of natural killer (NK) cells^50^ and the non-neutralizing antibody-dependent cellular cytotoxicity (ADCC) pathway^51^ may as the potential mechanisms of action by which the VLPs vaccine confers complete protection against lethal challenge from historical strains in mice. A limitation of our study is the lack of further exploration into these relevant immune mechanisms.

In this study, we confirmed and extended the above results by describing the short-term cellular responses induced by mosaic VLPs after immunizing mice. In the next step, we will further determine whether mosaic VLPs can induce an efficient and long-term immune response, along with the mechanisms involved. In summary, our work supported the development of VLPs vaccines based on mosaic genetic algorithms for combating influenza viruses. Here, we designed a universal influenza A virus vaccine and demonstrated that mosaic VLPs vaccination induced highly cross-reactive antibody and T-cell responses that protected against in vivo influenza virus challenges with contemporary and historical strains in mice. These data encourage further exploration of multivalent mosaic VLPs-based influenza virus vaccines that induce broad protective immune responses by targeting multiple antigens.

## Methods

### Ethics statement

This study was carried out in strict accordance with the recommendation in the Guide for the Care and Use of Laboratory Animals of the Ministry of Science and Technology of the People’s Republic of China. All experiments were approved by the Office of Research Protection’s Institutional Animal Care and Use Committee and Institutional Review Board at Sun Yat-sen University (approval number: 2022-B013). All experiments involving live viruses and animals were housed in negative-pressure isolators with HEPA filters in biosafety level 2 (BSL2) animal facilities at the Center for Disease Control and Prevention of Southern Military Theatre by the institutional biosafety manual.

### VLP production

Hemagglutinin and neuraminidase segment sequences of all human H1N1 and H3N2 from 2009 to 2021 used in this study were collected from Global Initiative on Sharing All Influenza Data (GISAID)and the National Center for Biotechnology Information (NCBI), including 55,514 sequences of H1, 74,410 sequences of H3, 50,135 sequences of N1, 74,403 sequences of N2. Then, a genetic algorithm was used to optimize each population in turn, in which new recombinants were generated and their epitope coverage was calculated and tested. Finally, four Mosaic recombinant antigen sequences were obtained, as described previously^18^.

H1m gene, H3m gene, N1m gene, and N2m gene, along with the A/Puerto Rico/8/34 (H1N1) matrix 1 protein (M1) gene, were cloned into the pFastBac expression vector immediately downstream of the polyhedron promoter, respectively. Then transformants were inserted into DH10Bac competent cells and, according to the Bac-to-Bac® manufacturer’s instructions harvested the recombinant baculoviruses (rBVs), named rBV-H1m, rBV-N1m, rBV-H3m, rBV-N2m and rBV-M1, respectively. Three separate recombinant baculoviruses containing HA、NA and M1 genes were used to produce the VLPs. After subculturing three times in Sf9 cells, the supernatant was centrifuged at 2576 × *g* at 4 °C for 30 min to remove cells. The collected supernatants containing VLPs were concentrated by ultracentrifugation at 11,0934 × *g* at 4 °C for 2 h. To further purify the particles, fractions containing the VLPs were subjected to a second sucrose gradient centrifugation. Then used PBS resuspend the precipitated particles at 4 °C, and VLPs were further purified through with discontinuous sucrose gradient (20%–30%–60%) at 11,0934 × *g* at 4 °C for 2 h. Then we assess the amount of residual baculovirus DNA in the preparation by Qubit Fluorometric Quantification.

### Influenza viruses

All seasonal influenza viruses, including A/Puerto Rico/8/34 (PR8) and A/Aichi/2/1968 (X-31), were kindly provided by the National Influenza Center of the Chinese Center for Disease Control and Prevention (China CDC).

All influenza viruses were grown in specific pathogen-free (SPF) embryonated eggs, and the chorioallantoic fluid was stored at −80 °C until use. Viral titers were determined and expressed as 50% tissue culture infective dose(s) (TCID50). All experimental studies with influenza viruses were conducted in biosafety level 2+ (BSL2+) facilities in compliance with the University of Sun Yat-sen Office of Biological Safety.

### Characterization of VLPs

VLPs were characterized using immunofluorescence assay (IFA), hemagglutination (HA), and neuraminidase (NA) activity assays. For IFA, HA antigens expression was detected using anti-influenza A H1N1 and H3N2 HA antibody (GeneTex, USA), NA antigens expression was detected using anti-influenza A H1N1 and H3N2 NA antibody (R&D, USA; Sino Biological, China) was used to confirm the expression of NA antigens, while anti-influenza M1 antibody (Abcam, USA) was used to confirm the expression of M1 antigens. Hemagglutination assay was performed to assess VLPs HA activity and used neuraminidase assay kit (Beyotime Biotechnology, China) to measure the neuraminidase activities of VLPs. HA-containing fractions were confirmed by HA assay. NA-containing fractions were confirmed by the Neuraminidase Assay Kit (Beyotime Biotechnology, China). The preparation of VLP was analyzed by negative-staining transmission electron microscope (TEM) to verify the morphological similarity of the production of the VLPs and the influenza virions.

### Immunofluorescence assay (IFA)

To determine the expression of the HA, NA, and M1 proteins of the rBVs, Sf9 cells were infected with the indicated rBVs at a multiplicity of infection (MOI) of 1. Following 48 h of incubation at 27 °C, Sf9 cells were fixed with cold acetone for 10 min. The cells were then incubated with the primary antibodies, including rabbit polyclonal antibody against the H1N1 and H3N2 HA protein (1:500, GTX127357 and GTX127363, GeneTex, USA) and H3N2 NA protein (1:500, 40017-T60, Sino Biological, China), sheep polyclonal antibody against the H1N1 NA protein (1:500, AF4858, R&D, USA) or M1 proteins (1:500, ab22396, Abcam, USA) for 1 h, respectively. After washing with PBS, the secondary antibodies, Alexa Fluor 488-conjugated goat anti-mouse IgG, Alexa Fluor 594-conjugated donkey anti-sheep IgG or Alexa Fluor 594-conjugated goat anti-rabbit IgG (1:500, ab150113, ab150180 and ab150080, Abcam, USA), were added to the cells and incubated at 37 °C for 1 h. The fluorescence signal was observed under a fluorescence microscope.

### Immunization and challenges of mice

To investigate the immunogenicity and efficacy of the VLPs in mice, 6-8-week-old specific pathogen-free (SPF) female BALB/c mice were divided into thirteen groups (*n* = 13). Mice were intramuscularly (i.m.) immunized twice with 1.5 μg of HA contents of each purified VLPs and quadrivalent inactivated influenza vaccine (QIV) in 100 μL of PBS at a 3-week interval. Mosaic VLPs were mixed evenly with 70,000 units /mL of IL-2 and 0.1% chitosan (Sigma-Aldrich, USA). Blood samples were collected at weeks 3 and 5 post vaccinations. Two weeks after the second immunization, all mice were intranasally challenged with 10 mouse-lethal doses 50% (MLD50) of the H1N1 and H3N2 influenza viruses in 50 μL of PBS. Three mice per group were euthanized and collected lungs at 4 dpi, and a TCID50 assay was performed to determine the virus titer. All mice were observed twice daily for clinical signs for 14 dpi to monitor body weight changes and mortality, 20% body weight loss was determined to be the humane intervention point, and mice reaching this endpoint were euthanized by inhalation of carbon dioxide and counted in mortality.

### Hemagglutination inhibition (HI) assay

To determine HI antibody titers in vaccinated mice, sera samples were initially diluted 1:10 in the receptor-destroying enzyme (RDE) (Denka Seiken, Japan) at 37 °C for 16 h and inactivated at 56 °C for 30 min. HI, the assay was performed using 1% chicken or guinea pig erythrocytes with 4 HA units of viruses according to standard protocols provided by the WHO. The HI titers were recorded as the highest serum dilution that showed completely inhibited hemagglutination.

### Neuraminidase inhibition (NI) assay

Neuraminidase inhibition (NI) was measured using enzyme-linked lectin assay (ELLA) in serum from immunized mice, as described previously^52^. Briefly, 96-well plates were coated with fetuin protein (25 μg/mL, 100 μL/well) in carbonate coating buffer, and incubated at 4 °C for more than 18 h. After washing and blocking, 96-well plates were incubated with serial dilutions of immune serum and influenza virus at 37 °C for 16 h. Upon 2 h after incubation with peroxidase-bound peanut lectin (1 μg/mL), 3,3,5,5-tetramethylbenzidine (TMB) was added, and OD 450 nm was measured to determine NI. The 50% inhibition percentage was defined as a serum concentration that produced at least 50% inhibition compared with the virus-only control.

### Microneutralization (MN) assay

To determine neutralization antibody titers in vaccinated mice, sera samples were treated with RDE at 37 °C for 16 h and inactivated at 56 °C for 30 min. Serially diluted sera reacted with 100 TCID50 influenza virus at 37 °C in 5% CO_2_ for 1 h, then added MDCK cells were in the virus-serum mixture and incubated at 37 °C in 5% CO_2_ for 18 h. After fixation with cold acetone, cells were incubated successively with an anti-influenza virus A antibody (1:4000, AB1074, Millipore, USA) and horseradish peroxidase-conjugated IgG (HRP-IgG) antibody (1:4000, FDG007, FDbio, China). TMB substrate was used to determine the microneutralization titers by measuring the OD_450_ nm value.

### Enzyme-linked immunosorbent assay (ELISA)

Highly binding polystyrene ELISA plates (Corning, USA) were coated with 5 μg/mL of the respective purified inactivated virus or VLPs in ELISA Coating Buffer (Solarbio, China) overnight at 4 °C. The following day, plates were blocked in 2% bovine serum albumin (BSA) in PBS with 0.05% Tween (PBST) for 60 min at RT and then incubated with diluted serum samples overnight at 4 °C. After incubation, plates were incubated with the respective secondary antibody, goat anti-mouse IgG, IgG1 and IgG2a (1:4000, 1036-05, 1071-05, and 1081-05, SouthernBiotech, USA) for 1 h at RT and added 3,3′,5,5′-Tetramethylbenzidine (TMB) developing solution (Solarbio, China) for 30 min. The plate was read at 450 nm on a plate reader (Thermo, USA).

### Enzyme-linked immunospot (ELISpot) assay

100 μL anti-mouse IFN-γ or IL-4 monoclonal antibody (15 μg/mL) (CT655-10 and CT657-10, U‐Cytech, Netherlands) was coated on ELISpot 96-well plates (Millipore, USA) incubated at 4 °C overnight, followed by blocking with 200 μL 1% bovine serum albumin (BSA) in PBS at room temperature for 2 h. Mouse splenocytes were isolated, 4 × 10^5^ cells/well were added to ELISpot 96-well plate, and then splenocytes were stimulated with inactivated PR8 or X-31 viruses at 37 °C in 5% CO_2_ for 28 h. After incubation, the splenocytes were removed, and biotin-conjugated anti-IFN-γ or anti-IL-4 antibody (CT655-10 and CT657-10, U‐Cytech, Netherlands) was added and subsequently incubated with streptavidin-ALP at room temperature for 1 h. Finally, after incubation in the 5-bromo-4chloro-3-indolyl-phosphate/nitro blue tetrazolium (BCIP/NBT) substrate solution for 20 min, the ELISpot Reader System (Mabtech, Sweden) was used to detect the number of spots.

### Flow cytometry and intracellular cytokine staining

The expression of cytokines was evaluated using flow cytometry for T cells in single-cell suspensions from the spleen. Splenocytes were stimulated in medium containing 10 μg/mL purified A/PR/8/1934 or A/Aichi/2/1968 virus for 2 h, and added BFA to incubated at 37 °C for 16 h. The cells were stained with murine antibodies for the phenotype (KO525-CD3, FITC-CD4, and AF700-CD8), and cytokine expression (PC7-IFN-γ, APC-IL-4, PE-TNF-α, and BV421-IL-2), and a dead or live cell dye ECD-Zombie Red was also used (100234, 100405, 100729, 505825, 504105, 506305, 503825 and 423110, Biolegend, USA). Finally, the cells were collected by CytoFLEX S Flow Cytometer (Beckman Coulter, USA), and the data were analyzed by CyExpert 2.4 (Beckman Coulter, USA) and FlowJo V10.8 (Tree Star, USA).

### Histological examination of the mice’s lung

Preparation of lung tissue of infected or uninfected mice by formalin fixation, paraffin embedding, and sectioning. Then the samples were stained with hematoxylin and eosin, respectively.

### Statistical analysis

GraphPad Prism 8 software was used to analyze all data. The statistical significance was determined by either one- or two-way ANOVA followed by a multiple comparison test. Data were expressed as the mean with standard error (SEM). *P* values < 0.05 was considered statistically significant.

### Reporting summary

Further information on research design is available in the Nature Research Reporting Summary linked to this article.

### Supplementary information

Reporting Summary

Supplementary Information

## Data Availability

The Mosaic vaccine designer algorithm used in this study is freely available at https://www.hiv.lanl.gov/content/sequence/MOSAIC/makeVaccine.html. All sequences used to create the Mosaic immunogens are freely available through the Influenza Virus Database at https://www.ncbi.nlm.nih.gov/genomes/FLU/Database/nph-select.cgi#mainform and https://gisaid.org. All other relevant data will be provided by the corresponding author upon request. Some data in this manuscript will be included in the patent application.
