# Supplementary material for: A mosaic influenza virus-like particles vaccine provides broad humoral and cellular immune responses against influenza A viruses
Source: NPJ Vaccines. 2023 Sep 7;8:132. doi: 10.1038/s41541-023-00728-5 (PMC10485063; doi:10.1038/s41541-023-00728-5)
Supplement: Supplementary file 1 — Reporting Summary [file 41541_2023_728_MOESM1_ESM.pdf]

nature portfolio

Corresponding author(s): Caijun Sun, Huacheng Yan, Yuelong Shu

Last updated by author(s): Jul 15, 2023

Reporting Summary

Nature Portfolio wishes to improve the reproducibility of the work that we publish. This form provides structure for consistency and transparency in reporting. For further information on Nature Portfolio policies, see our Editorial Policies and the Editorial Policy Checklist.

Statistics

For all statistical analyses, confirm that the following items are present in the figure legend, table legend, main text, or Methods section.

n/a	Confirmed	
☐	☒	The exact sample size (*n*) for each experimental group/condition, given as a discrete number and unit of measurement
☐	☒	A statement on whether measurements were taken from distinct samples or whether the same sample was measured repeatedly
☐	☒	The statistical test(s) used AND whether they are one- or two-sided<br>*Only common tests should be described solely by name; describe more complex techniques in the Methods section.*
☒	☐	A description of all covariates tested
☒	☐	A description of any assumptions or corrections, such as tests of normality and adjustment for multiple comparisons
☐	☒	A full description of the statistical parameters including central tendency (e.g. means) or other basic estimates (e.g. regression coefficient) AND variation (e.g. standard deviation) or associated estimates of uncertainty (e.g. confidence intervals)
☒	☐	For null hypothesis testing, the test statistic (e.g. *F*, *t*, *r*) with confidence intervals, effect sizes, degrees of freedom and *P* value noted<br>*Give P values as exact values whenever suitable.*
☒	☐	For Bayesian analysis, information on the choice of priors and Markov chain Monte Carlo settings
☒	☐	For hierarchical and complex designs, identification of the appropriate level for tests and full reporting of outcomes
☒	☐	Estimates of effect sizes (e.g. Cohen's *d*, Pearson's *r*), indicating how they were calculated

Our web collection on statistics for biologists contains articles on many of the points above.

Software and code

Policy information about availability of computer code

Data collection	Open data were collection through the GISAID at https://gisaid.org/ and Influenza Virus Database of NCBI at https://www.ncbi.nlm.nih.gov/genomes/FLU
Data analysis	Data was analyzed using the following software: Mafft-win, Edit Plus 4, AliView, BioAider v1.314, BioEdit v7.2.5, Clustal W, Mega 7, FigTree v1.4.4, Curve Expert 1.4, DNASTAR.Lasergene.v7.1, FlowJo 10.8, GraphPad Prism 8, ChimeraX

For manuscripts utilizing custom algorithms or software that are central to the research but not yet described in published literature, software must be made available to editors and reviewers. We strongly encourage code deposition in a community repository (e.g. GitHub). See the Nature Portfolio guidelines for submitting code & software for further information.

Data

Policy information about availability of data

All manuscripts must include a data availability statement. This statement should provide the following information, where applicable:

- Accession codes, unique identifiers, or web links for publicly available datasets
- A description of any restrictions on data availability
- For clinical datasets or third party data, please ensure that the statement adheres to our policy

The mosaic vaccine designer algorithm used in this study is freely available at https://www.hiv.lanl.gov/content/sequence/MOSAIC/makeVaccine.html. All

sequences used to create the mosaic immunogens are freely available through the GISAID at https://gisaid.org/ and Influenza Virus Database of NCBI at https://www.ncbi.nlm.nih.gov/genomes/FLU. All other data will be provided by the corresponding author upon request.

Research involving human participants, their data, or biological material

Policy information about studies with human participants or human data. See also policy information about sex, gender (identity/presentation), and sexual orientation and race, ethnicity and racism.

Reporting on sex and gender	*Use the terms sex (biological attribute) and gender (shaped by social and cultural circumstances) carefully in order to avoid confusing both terms. Indicate if findings apply to only one sex or gender; describe whether sex and gender were considered in study design; whether sex and/or gender was determined based on self-reporting or assigned and methods used. Provide in the source data disaggregated sex and gender data, where this information has been collected, and if consent has been obtained for sharing of individual-level data; provide overall numbers in this Reporting Summary. Please state if this information has not been collected. Report sex- and gender-based analyses where performed, justify reasons for lack of sex- and gender-based analysis.*
Reporting on race, ethnicity, or other socially relevant groupings	*Please specify the socially constructed or socially relevant categorization variable(s) used in your manuscript and explain why they were used. Please note that such variables should not be used as proxies for other socially constructed/relevant variables (for example, race or ethnicity should not be used as a proxy for socioeconomic status). Provide clear definitions of the relevant terms used, how they were provided (by the participants/respondents, the researchers, or third parties), and the method(s) used to classify people into the different categories (e.g. self-report, census or administrative data, social media data, etc.) Please provide details about how you controlled for confounding variables in your analyses.*
Population characteristics	*Describe the covariate-relevant population characteristics of the human research participants (e.g. age, genotypic information, past and current diagnosis and treatment categories). If you filled out the behavioural & social sciences study design questions and have nothing to add here, write "See above."*
Recruitment	*Describe how participants were recruited. Outline any potential self-selection bias or other biases that may be present and how these are likely to impact results.*
Ethics oversight	*Identify the organization(s) that approved the study protocol.*

Note that full information on the approval of the study protocol must also be provided in the manuscript.

Field-specific reporting

Please select the one below that is the best fit for your research. If you are not sure, read the appropriate sections before making your selection.

☒ Life sciences ☐ Behavioural & social sciences ☐ Ecological, evolutionary & environmental sciences

For a reference copy of the document with all sections, see nature.com/documents/nr-reporting-summary-flat.pdf

Life sciences study design

All studies must disclose on these points even when the disclosure is negative.

Sample size	We used sample sizes of 5-8 animals per group. The number of animals is clearly described in the methods and figure legends. A sample size of 5 is sufficient to reach statistical significance. Experiments assessing virus titres in the lungs of mice only contained 3 mice/group due to the complexity of harvesting organs from multiple groups of mice.
Data exclusions	No data were excluded.
Replication	In many cases the experiments were successfully replicated.
Randomization	No randomization needed.
Blinding	Investigators were not blinded in our studies. The investigators who designed the experiments also performed the experiments so blinding was not possible

Reporting for specific materials, systems and methods

We require information from authors about some types of materials, experimental systems and methods used in many studies. Here, indicate whether each material, system or method listed is relevant to your study. If you are not sure if a list item applies to your research, read the appropriate section before selecting a response.

Materials & experimental systems

n/a	Involved in the study	
☐	☒	Antibodies
☐	☒	Eukaryotic cell lines
☒	☐	Palaeontology and archaeology
☐	☒	Animals and other organisms
☒	☐	Clinical data
☒	☐	Dual use research of concern
☒	☐	Plants

Methods

n/a	Involved in the study	
☒	☐	ChIP-seq
☐	☒	Flow cytometry
☒	☐	MRI-based neuroimaging

Antibodies

Antibodies used: Influenza A virus H1N1 HA (Hemagglutinin) antibody (GTX127357; Genetex),Influenza A virus H3N2 HA (Hemagglutinin) antibody (GTX127363; Genetex), Influenza A Virus H1N1 Neuraminidase Antibody (AF4858; R&D), Influenza A H3N2 Neuraminidase / NA Antibody (40017-T60, Sino Biological), Anti-Matrix protein 1 antibody [GA2B] (ab22396; Abcam), Alexa Fluor 488 conjugated goat anti-mouse IgG, Alexa Fluor 594 conjugated donkey anti-sheep IgG and Alexa Fluor 594 conjugated goat anti-rabbit IgG (ab150113, ab150180 and ab150080, Abcam), Goat anti-mouse IgG(H+L) (1036-05; SouthernBiotech), Goat anti-mouse IgG1 (1071-05; SouthernBiotech),Goat anti-mouse IgG2a (1081-05; SouthernBiotech), Brilliant Violet 510™ anti-mouse CD3 Antibody (100234; Biolegend), FITC anti-mouse CD4 Antibody (100405; Biolegend), Alexa Fluor® 700 anti-mouse CD8a Antibody (100729; Biolegend), PE/Cyanine7 anti-mouse IFN-γ Antibody (505825; Biolegend), APC anti-mouse IL-4 Antibody (504105; Biolegend), PE anti-mouse TNF-α Antibody (506305; Biolegend), Brilliant Violet 421™ anti-mouse IL-2 Antibody (503825; Biolegend), Zombie Red™ Fixable Viability Kit (423110; Biolegend)

Validation: All antibodies were supplied by commercial vendors or repositories and include quality control assessments

Eukaryotic cell lines

Policy information about cell lines and Sex and Gender in Research

Cell line source(s): SF9 and MDCK cells were all obtained from ATCC.

Authentication: None of the cell lines were authenticated

Mycoplasma contamination: None of the cell lines were assessed for mycoplasma contamination.

Commonly misidentified lines (See ICLAC register): No commonly misidentified cell lines were used in this study.

Animals and other research organisms

Policy information about studies involving animals; ARRIVE guidelines recommended for reporting animal research, and Sex and Gender in Research

Laboratory animals: Female BALB/C mice ages 6-8 weeks were purchased from Zhuhai BesTest Bio-Tech Co,.Ltd (License number, SYXK (Yue) 2019-0100)

Wild animals: No wild animals were used in this study.

Reporting on sex: We used all female mice for this study and did not set up different sex groups for comparative studies.

Field-collected samples: No field-collected samples were used in this study.

Ethics oversight: All experiments were approved by the Office of Research Protection's Institutional Animal Care and Use Committee and Institutional Review Board at Sun Yat-sen University (approval number: 2022-B013). All experiments involving live viruses and animals were housed in negative-pressure isolators with HEPA filters in biosafety level 2 (BSL2) animal facilities at Center for Disease Control and Prevention of Southern Military Theatre by the institutional bio-safety manual.

Note that full information on the approval of the study protocol must also be provided in the manuscript.

Flow Cytometry

Plots

Confirm that:

- ☐ The axis labels state the marker and fluorochrome used (e.g. CD4-FITC).
- ☐ The axis scales are clearly visible. Include numbers along axes only for bottom left plot of group (a 'group' is an analysis of identical markers).
- ☒ All plots are contour plots with outliers or pseudocolor plots.
- ☒ A numerical value for number of cells or percentage (with statistics) is provided.

Methodology

Sample preparation	Spleens were disrupted by gentle mechanical disruption using the plunger of a 1 mL syringe and passed through 40 µm cell strainer to obtained single cells suspension. Red blood cells were lysed with RBC lysis buffer and washed with PBS.
Instrument	CytoFLEX S Flow Cytometer (Beckman Coulter, USA)
Software	CyExpert 2.4 software (Beckman Coulter, USA). Sequential gating was performed using FlowJo V10.8 (Tree Star, USA).
Cell population abundance	Sorting of the cells or enrichment of one single cell type were not performed in this study.
Gating strategy	Lymphocytes were first gated on forward and side scatter, followed by singlet gating and then live cells. Activated cells of interest were defined as CD4+ T or CD8+ T cells that were positive for cytokine staining (IFN-γ, IL-4, TNF-α and IL-2).

☒ Tick this box to confirm that a figure exemplifying the gating strategy is provided in the Supplementary Information.
