# Supplementary material for: A mosaic influenza virus-like particles vaccine provides broad humoral and cellular immune responses against influenza A viruses
Source: NPJ Vaccines. 2023 Sep 7;8:132. doi: 10.1038/s41541-023-00728-5 (PMC10485063; doi:10.1038/s41541-023-00728-5)
Supplement: Supplementary file 2 — Supplementary Information [file 41541_2023_728_MOESM2_ESM.pdf]

**Supplementary Data - The mosaic sequences**

>H1m
MKQGKTKATKMKAILVVLLYTFATANADTLCIGYHANNSTDTVDTVLEKNVTVTHSVNLL
EDKHNGKLCKLRGVAPLHLGKCNIAGWILGNPECESLSTASSWSYIVETSSSDNGTCYPGD
FIDYEELREQLSSVSSFERFEIFPKTSSWPNHDSNKGVTAACPHAGAKSFYKNLIWLVKKGNS
YPKLSKSYINDKGKEVLVLWGIHHPSTSADQQSLYQNADAYVFVGTSRYSKKFKPEIAIRPK
VRDQEGRMNYYWTLVEPGDKITFEATGNLVVPRYAFAMERNAGSGIIISDTPVHDCNTTCQ
TPKGAINTSLPFQNIHPITIGKCPKYVKSTKLRLATGLRNVPSIQSRGLFGAIAGFIEGGWTG
MVDGWYGYHHQNEQGSGYAADLKSTQNAIDKITNKVNSVIEKMNTQFTAVGKEFNHLE
KRIENLNKKVDDGFLDIWTYNAELLVLLENERTLDYHDSNVKNLYEKVRSQLKNNAKEIG
NGCFEFYHKCDNTCMESVKNGTYDYPKYSEEAKLNREEIDGVKLESTRIYQILAIYSTVASSL
VLVVSLGAISFWMCSNGSLQCRICI*

>H3m
MKTIIALSYILCLVFAQKIPGNDNSTATLCLGHHAVPNGTIVKTITNDRIEVTNATELVQNSSI
GEICDSPHQILDGENCTLIDALLGDPQCDGFQNKKWDLFVERSKAYSNCYPYDVPDYASLR
SLVASSGTLEFNNESFNWTGVTQNGTSSACIRRSSSSFFSRLNWLTHLNYTYPALNVTMPNN
EQFDKLYIWGVHHPGTDKDQIFLYAQSSGRITVSTKRSQQAVIPNIGSRPRIRDIPSRISIYWTI
VKPGDILLINSTGNLIAPRGYFKIRSGKSSIMRSDAPIGKCKSECITPNGSIPNDKPFQNVNRIT
YGACPRYVKQSTLKLATGMRNVPEKQTRGIFGAIAGFIENGWEGMVDGWYGFRHQNSEG
RGQAADLKSTQAAIDQINGKLNRLIGKTNEKFHQIEKEFSEVEGRIQDLEKYVEDTKIDLWS
YNAELLVALENQHTIDLTDSEMNKLFEKTKKQLRENAEDMGNGCFKIYHKCDNACIGSIR
NGTYDHNVYRDEALNNRFQIKGVELKSGYKDWILWISFAISCFLLCVALLGFIMWACQKG
NIRCNICI*

>N1m
MNPNQKIITIGSVCMTIGMANLILQIGNIISIWVSHSIQIGNQSQIETCNQSVITYENNTWVN
QTYVNISNTNFAAGQSVVSVKLAGNSSLCPVSGWAIYSKDNSVRIGSKGDVFVIREPFISCSP
LECRTFFLTQGALLNDKHSNGTIKDRSPYRTLMSCPIGEVPSPYNSRFESVAWSASACHDGI
NWLTIGISGPDSGAVAVLKYNGIITDTIKSWRNNILRTQESECACVNGSCFTIMTDGPSDGQ
ASYKIFRIEKGKIVKSVEMNAPNYHYEECSCYPDSSEITCVCRDNWHGSNRPWVSFNQNLE
YQIGYICSGVFGDNPRPNDKTGSCGPVSSNGANGVKGFSFKYGNGVWIGRTKSISSRKGFE
MIWDPNGWTGTDNNFSIKQDIVGINEWSGYSGSFVQHPELTGLDCIRPCFWVELIRGRPEE
NTIWTSGSSISFCGVNSDTVGWSWPDGAELPFTIDK*
>N2m

MNPNQKIITIGSVSLTISTICFFMQIAILITTVTLHFKQYEFNSPPNNQVMLCEPTIIERNITEIV
YLTNTTIEKEICPKPAEYRNWSKPQCGITGFAPFSKDNSIRLSAGGDIWVTREPYVSCDPDKC
YQFALGQGTTLNNVHSNNTVRDRTPYRTLLMNELGVPFHLGTKQVCIAWSSSSCHDGKA
WLHVCITGDDKNATASFIYNGRLVDSVVSWSKDILRTQESECVCINGTCTVVMTDGNATG
KADTKILFIEEGKIVHTSKLSGSAQHVEECSCYPRYPGVRCVCRDNWKGSNRPIVDINIKDH
SIVSSYVCSGLVGDTPRKNDSSSSSHCLDPNNEEGGHGVKGWAFDDGNDVWMGRTINETS
RLGYETFKVVEGWSNPKSKLQINRQVIVDRGDRSGYSGIFSVEGKSCINRCFYVELIRGRKEE
TEVLWTSNSIVVFCGTSGTYGTGSWPDGADLNLMPI*

Supplementary Table 1. The HA and NA proteins of strains used in this study were analyzed for identity and similarity to mosaic HA and NA sequences.

| Antigen | Identity | Similarity | Antigen | Identity | Similarity |
|---|---|---|---|---|---|
| HAm-H1/NAm-N1 | 100/100 | 100/100 | HAm-H3/NAm-N2 | 100/100 | 100/100 |
| A/Michigan/45/2015 | 98.06/98.93 | 98.41/99.36 | A/Kansas/14/2017 | 98.06/98.29 | 98.23/99.15 |
| A/Victoria/2570/2019 | 95.94/96.38 | 97.35/97.65 | A/Hong Kong/4801/2014 | 98.06/98.72 | 98.06/99.36 |
| A/Guangdong-Maonan/ SWL1536/2019 | 96.64/96.80 | 97.70/97.87 | A/Hong Kong/2671/2019 | 97.17/98.72 | 97.88/99.15 |
| A/California/7/2009 | 98.76/98.08 | 99.12/99.15 | A/Cambodia/E0826360/2020 | 97.17/97.44 | 97.88/98.08 |
| A/Brisbane/02/2018 | 96.82/98.08 | 97.70/98.72 | A/Texas/50/2012 | 98.41/98.51 | 99.12/99.36 |
| A/California/04/2009 | 98.94/98.08 | 99.29/99.15 | A/Singapore/INFIMH-16-0019/2016 | 98.76/99.36 | 98.94/99.79 |
| A/Hunan/42443/2015 | 78.27/90.19 | 87.10/93.82 | A/Switzerland/971529/2013 | 98.41/98.93 | 98.94/99.36 |
| A/Puerto Rico/8/1934 | 43.82/48.40 | 51.41/53.73 | A/Aichi/2/1968 | 86.04/84.65 | 92.05/90.41 |

# Qubit™ 4
## Assay Run Results

Thermo Fisher SCIENTIFIC

| | |
|---|---|
| **Software Version** | 2.19 |
| **Run ID** | 2023-06-26_103821 |
| **Assay Name** | dsDNA High Sensitivity |
| **Extended Low Range (ng/mL)** | 0.5-1.0 |
| **Core Range (ng/mL)** | 1.0-500.0 |
| **Extended High Range (ng/mL)** | 500.0-600.0 |
| **Excitation** | Blue (430-495 nm) |
| **Emission** | Green (510-580 nm) |
| **Std 1 RFU** | 38.47 |
| **Std 2 RFU** | 25366.31 |
| **Std 3 RFU** | - |

| Test Name | Test Date | Sample Volume (μL) | Dilution Factor | Qubit Tube Conc. | Original Sample Con. | Green RFU |
|---|---|---|---|---|---|---|
| Sample_#230626-104532 | 26/06/2023 10:45:32 AM | 2 | 100 | * | * | 38.54 |
| Sample_#230626-104456 | 26/06/2023 10:44:56 AM | 2 | 100 | 0.65 ng/mL | 0.0001 μg/μL | 69.70 |

* Out of range

Supplementary Figure 1. The amount of residual baculovirus DNA in the mosaic VLPs by Qubit Fluorometric Quantification. The concentration of dsDNA in VLP-1-1 < 0.5 ng/mL, shown as "* Out of range"; The concentration of dsDNA in VLP-3-2 was 0.65 ng/mL. (Sample_#230626-104456 is VLP-1-1; Sample_#230626-104532 is VLP-3-2).

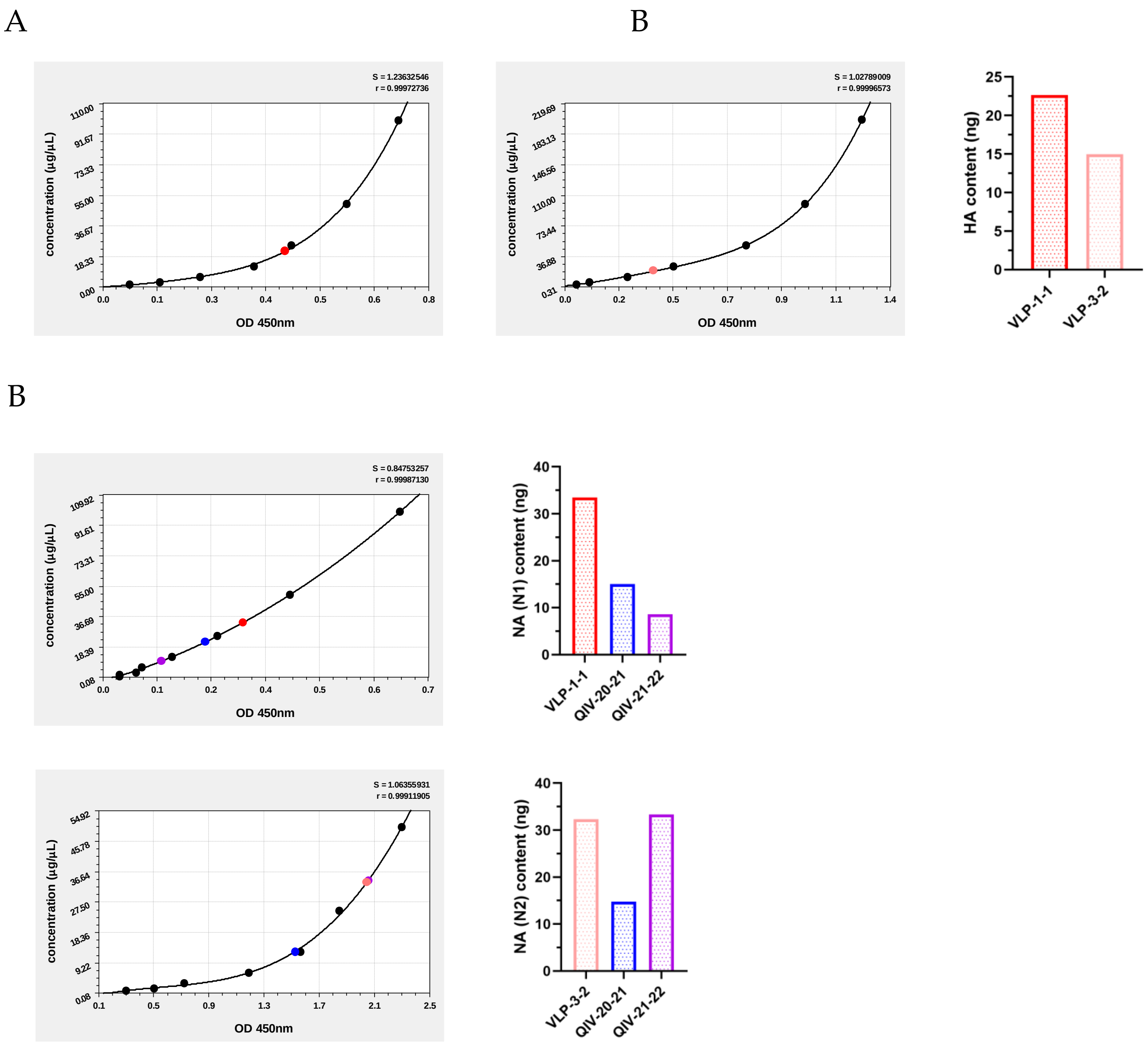


Supplementary Figure 2. Quantification of HA and NA in mosaic VLPs and QIV by ELISA. (A) Using decreasing concentrations of recombinant HA protein as standard protein, measured HA content in 1μg VLPs. A standard curve was generated for HA concentration based on absorbance values from standard protein (black dotted lines), the goodness of fit statistic for the standard curve is also displayed (polynomial fit). The concentration of NA in the mosaic VLPs was extrapolated from the curve (red and pink dot). (B) Using decreasing concentrations of recombinant NA protein as standard protein, measured NA content in 0.15μg HA of mosaic VLPs and QIV. A standard curve was generated for NA concentration based on absorbance values from standard protein (black dotted lines), the goodness of fit statistic for the standard curve is also displayed (polynomial fit). The concentration of NA in the mosaic VLPs was extrapolated from the curve (red, pink, blue and purple dot).

A

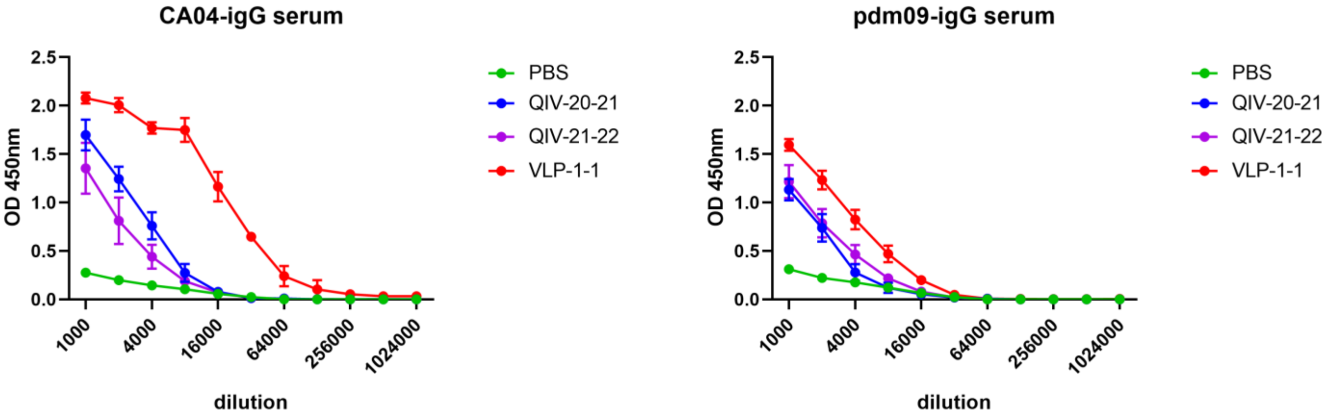


B

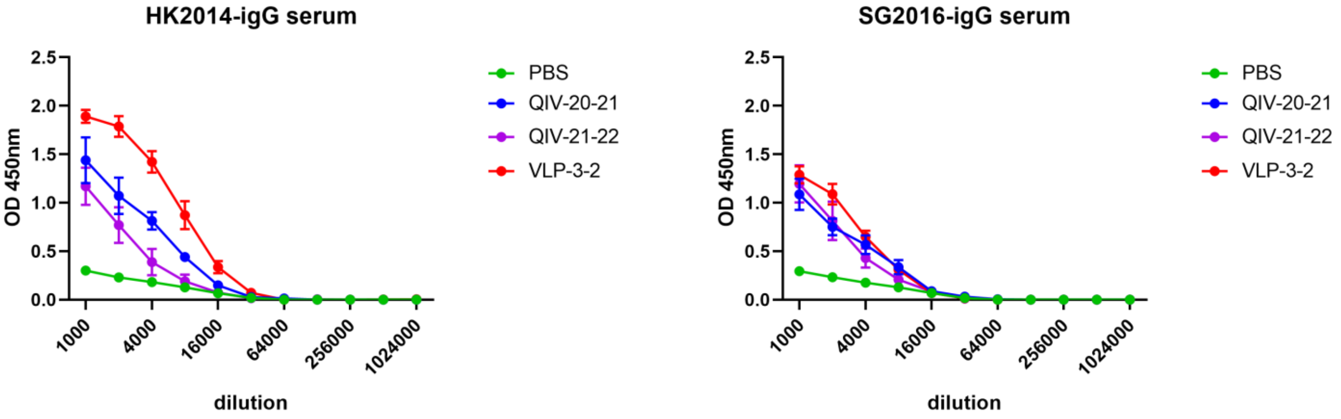


Supplementary Figure 3. Mosaic VLPs immunization elicits higher antibody titers in mice against H1 and H3 representative viruses. Antibody binding was determined by ELISA assay. (A) ELISA against H1 representative strains A/California/04/2009 (CA04) and A/California/7/2009 (pdm09). (B) ELISA against H3 representative strains A/Hong_Kong/4801/2014 (HK2014) and A/Singapore/INFIMH-16-0019/2016 (SG2016).

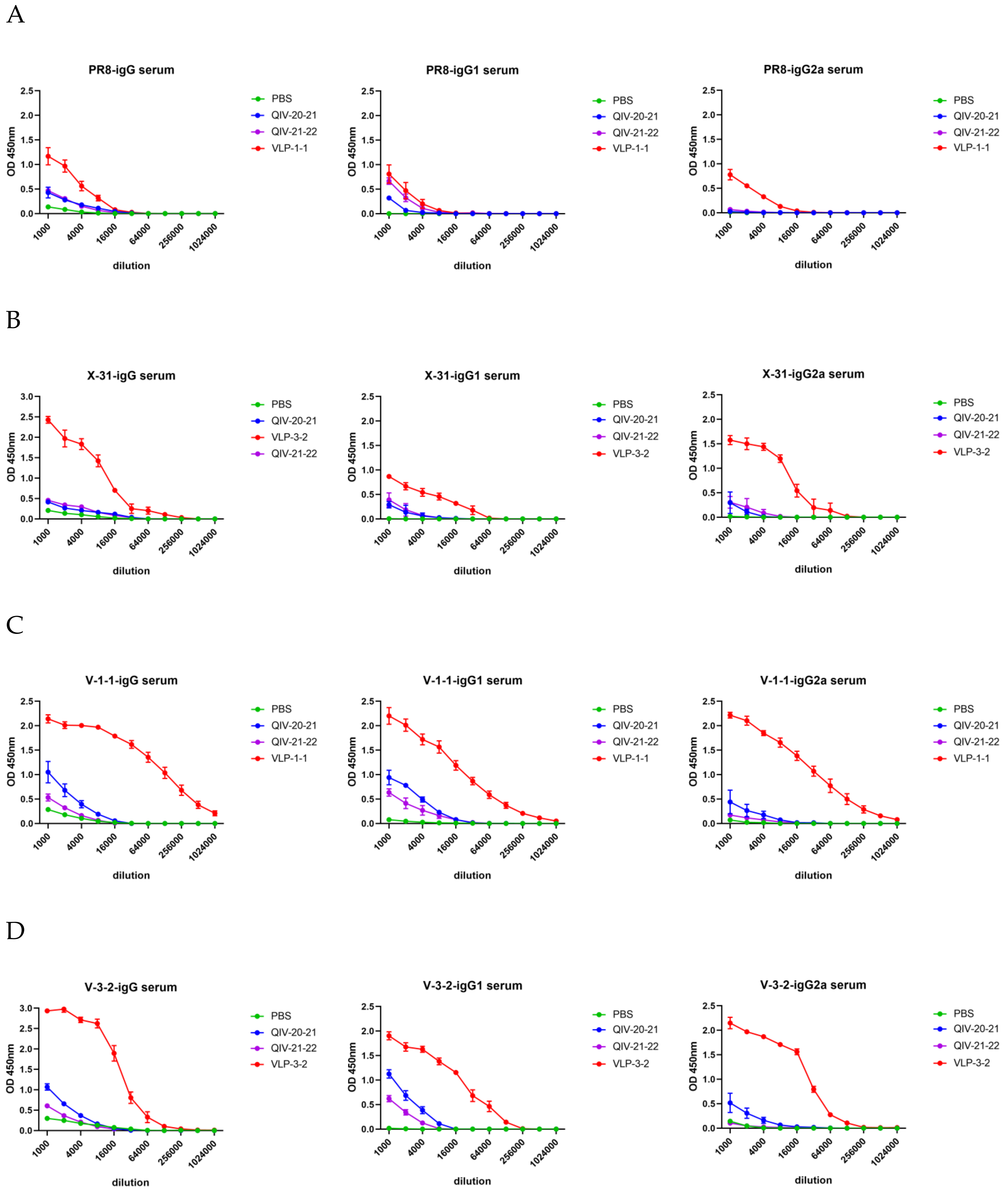


Supplementary Figure 4. Antigenicity of mosaic VLPs and QIVs. Antibody binding was determined by ELISA assay. ELISA against (A) A/Puerto Rico/8/1934 (PR8), (B) A/Aichi/2/1968 (X-31), (C) mosaic H1N1 VLP and (D) mosaic H3N2 VLP.

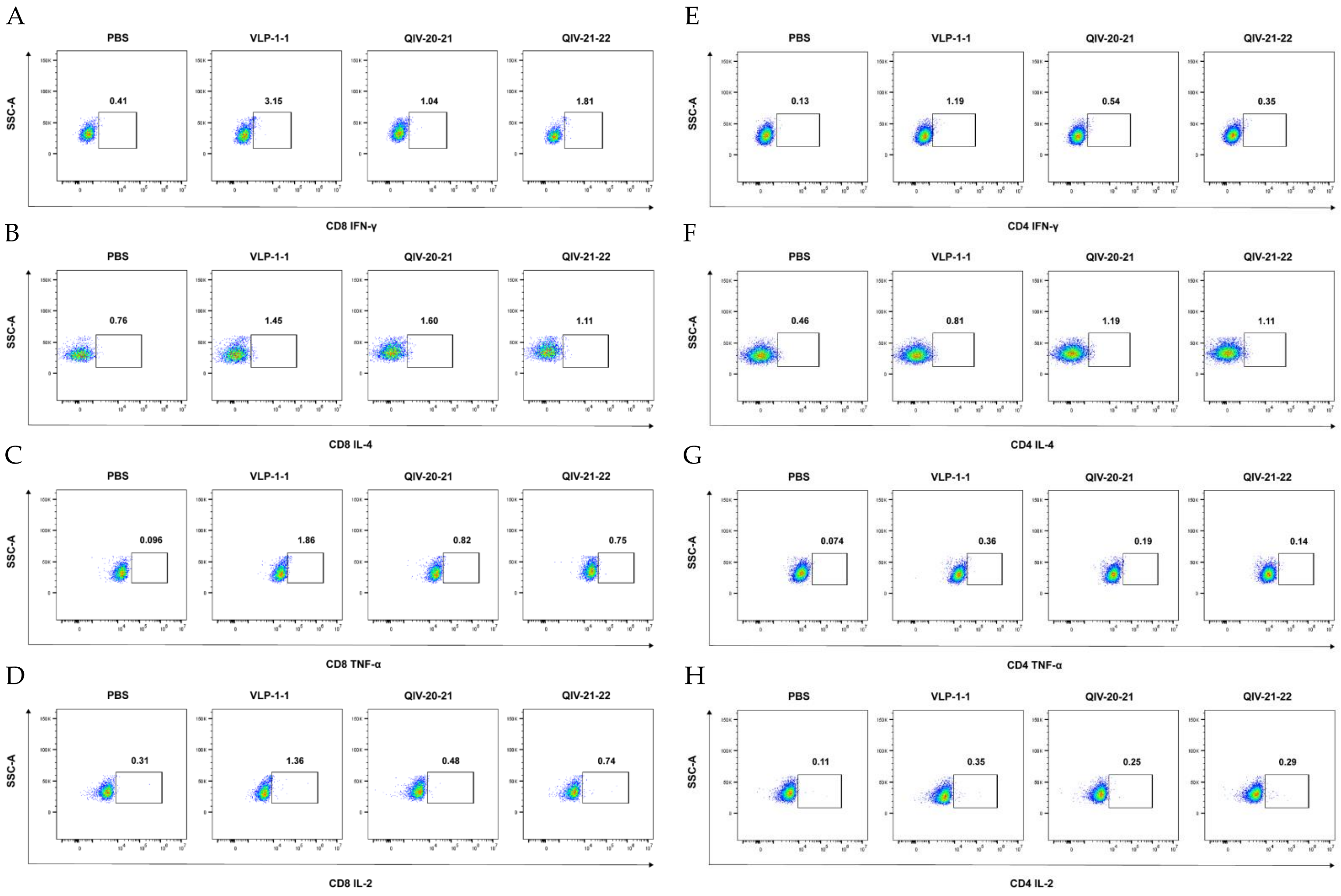


Supplementary Figure 5. Representative flow images of CD8$^+$ IFN-$\gamma^+$ T cells (A), CD8$^+$ IL-4$^+$ T cells (B), CD8$^+$ TNF-$\alpha^+$ T cells (C), CD8$^+$ IL-2$^+$ T cells (D), CD4$^+$ IFN-$\gamma^+$ T cells (E), CD4$^+$ IL-4$^+$ T cells (F), CD4$^+$ TNF-$\alpha^+$ T cells (G), and CD4$^+$ IL-2$^+$ T cells (H) generated by virus A/PR/8/1934 (PR8) stimulation.

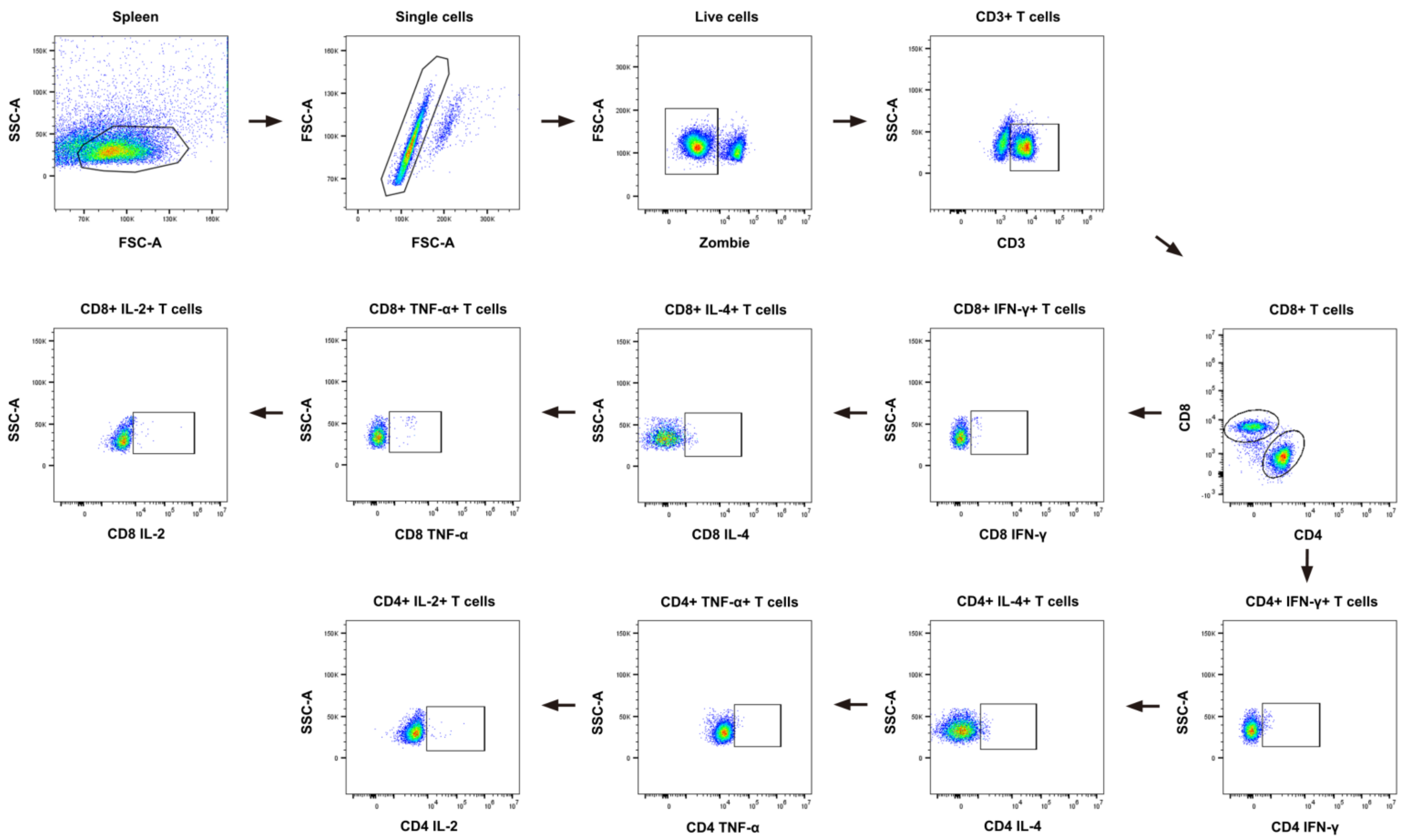


Supplementary Figure 6. Flow cytometric gating strategies of CD8$^+$ IFN-$\gamma^+$, CD8$^+$ IL-4$^+$, CD8$^+$ TNF-$\alpha^+$, CD8$^+$ IL-2$^+$, and CD4$^+$ IFN-$\gamma^+$, CD4$^+$ IL-4$^+$, CD4$^+$ TNF-$\alpha^+$, CD4$^+$ IL-2$^+$ T cells.

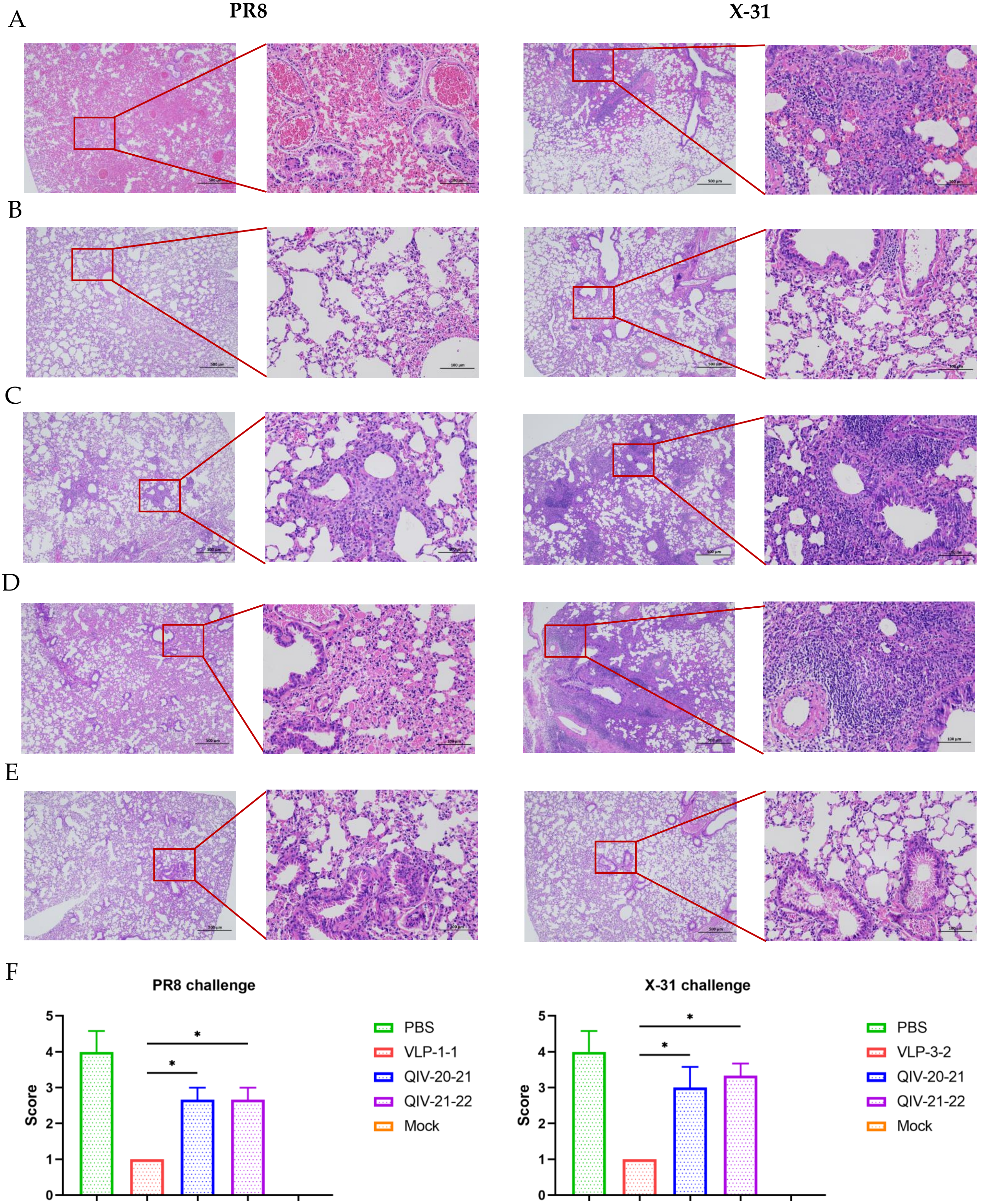


Supplementary Figure 7. Lung histology of challenged mice. Mice were challenged with A/Puerto Rico/8/34 (PR8) and A/Aichi/2/1968 (X-31) viruses at week 5 following immunization with vaccines PBS (A) , mosaic VLPs (B), QIV20-21 (C), and QIV21-22 (D). Non-challenged control mice (E). At days 4 post-infection, the mice were euthanized and lungs were harvested. Lungs were inflated with 10% formalin, fixed and processed for paraffin embedding, and 5-mm sections stained for H&E. Observations were made at 40× and 200× magnification. (F) Lungs were scored according to severity of overall lesions, where 0 indicates no abnormalities detected, 1 indicates minimal abnormalities (5 to 20% lung area), 2 indicates mild abnormalities (20 to 40% lung area), 3 indicates moderate abnormalities (40 to 60% lung area), 4 indicates marked abnormalities (60 to 80% lung area), and 5 indicates severe abnormalities (80 to 100% lung area). (n = 3; one-way ANOVA with Tukey multiple comparison), *$p < 0.05$, **$p < 0.01$.

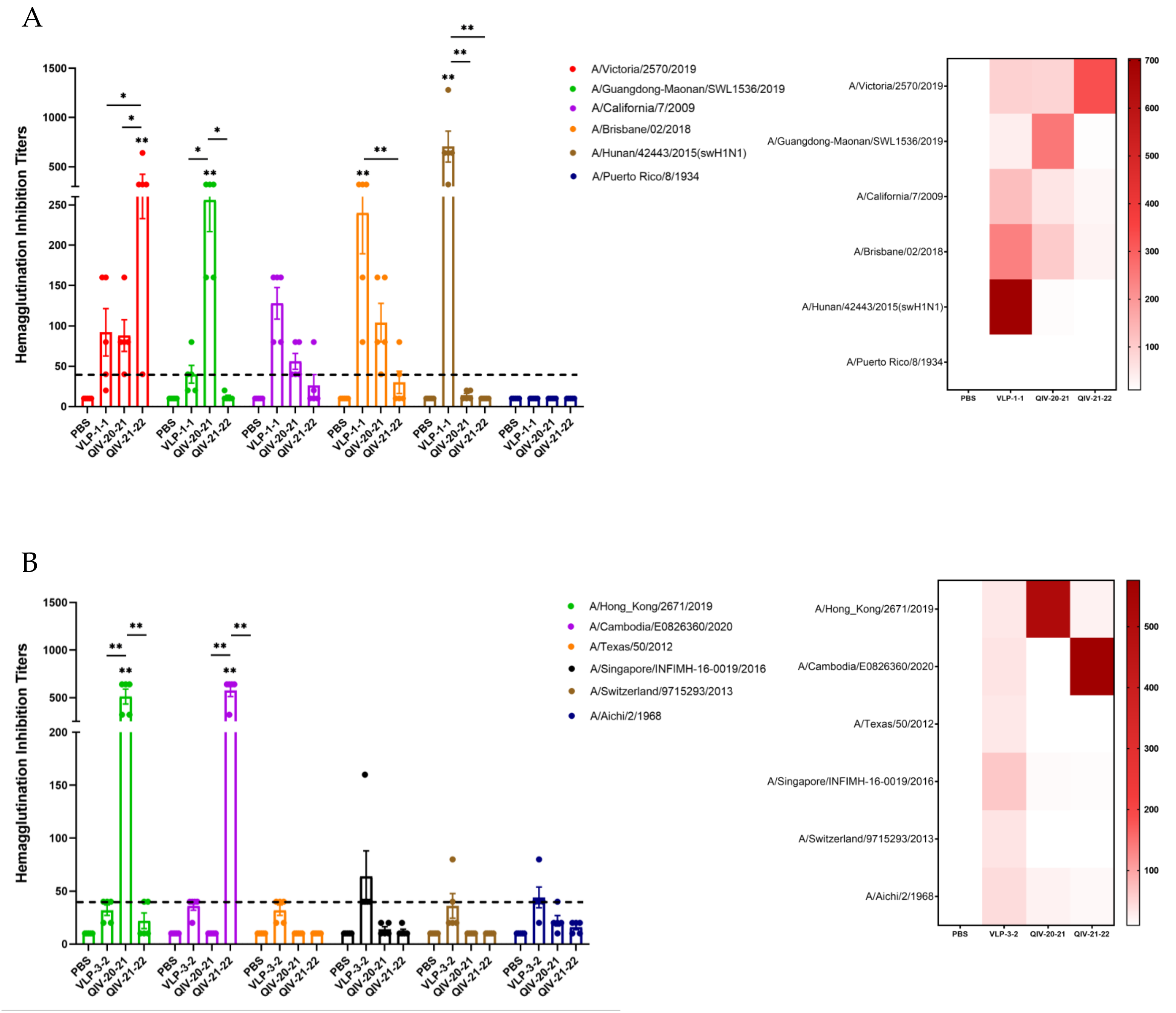


Supplementary Figure 8. Mosaic VLPs immunization elicits robust cross-reactive antibody responses in mice against H1 and H3 representative viruses. BALB/c mice were vaccinated with VLPs and compared to QIV-vaccinated mice. The HI antibody response to H1 (A) or H3 (B) representative strains was determined by HI assay in mice immunized with prime immunization. A heat map of HI titers was further constructed to better visualize cross-reactive antibody response for each vaccine. Data are presented as the mean with standard error (SEM ) (n = 5; two-way ANOVA with Tukey multiple comparison), *$p < 0.05$, **$p < 0.01$.
